# Extracting Reproducible Time-Resolved Resting State Networks Using Dynamic Mode Decomposition

**DOI:** 10.3389/fncom.2019.00075

**Published:** 2019-10-31

**Authors:** James M. Kunert-Graf, Kristian M. Eschenburg, David J. Galas, J. Nathan Kutz, Swati D. Rane, Bingni W. Brunton

**Affiliations:** ^1^Pacific Northwest Research Institute, Seattle, WA, United States; ^2^Department of Bioengineering, University of Washington, Seattle, WA, United States; ^3^Department of Applied Math, University of Washington, Seattle, WA, United States; ^4^Department of Radiology, University of Washington, Seattle, WA, United States; ^5^Department of Biology, University of Washington, Seattle, WA, United States

**Keywords:** resting state network (RSN), dynamic mode decomposition (DMD), RS-fMRI, human connectome project (HCP), individualized networks, network dynamics

## Abstract

Resting state networks (RSNs) extracted from functional magnetic resonance imaging (fMRI) scans are believed to reflect the intrinsic organization and network structure of brain regions. Most traditional methods for computing RSNs typically assume these functional networks are static throughout the duration of a scan lasting 5–15 min. However, they are known to vary on timescales ranging from seconds to years; in addition, the dynamic properties of RSNs are affected in a wide variety of neurological disorders. Recently, there has been a proliferation of methods for characterizing RSN dynamics, yet it remains a challenge to extract reproducible time-resolved networks. In this paper, we develop a novel method based on dynamic mode decomposition (DMD) to extract networks from short windows of noisy, high-dimensional fMRI data, allowing RSNs from single scans to be resolved robustly at a temporal resolution of seconds. After validating the method on a synthetic dataset, we analyze data from 120 individuals from the Human Connectome Project and show that unsupervised clustering of DMD modes discovers RSNs at both the group (gDMD) and the single subject (sDMD) levels. The gDMD modes closely resemble canonical RSNs. Compared to established methods, sDMD modes capture individualized RSN structure that both better resembles the population RSN and better captures subject-level variation. We further leverage this time-resolved sDMD analysis to infer occupancy and transitions among RSNs with high reproducibility. This automated DMD-based method is a powerful tool to characterize spatial and temporal structures of RSNs in individual subjects.

## 1. Introduction

Resting state networks (RSNs) comprise distinct regions of the brain that exhibit synchronous low-frequency (<0.1 Hz) temporal fluctuations in the absence of explicit tasks (Raichle et al., 2001; Damoiseaux et al., 2006; Fox and Raichle, 2007). They are most commonly detected using blood-oxygen level-dependent (BOLD) resting state functional magnetic resonance imaging (rs-fMRI), where a series of T2 or T2* weighted images of the brain are acquired repeatedly over the duration of the scan (5–15 min) (Lowe et al., 1998; Van Den Heuvel and Pol, 2010). The data-driven extraction of RSNs from these noisy and high-dimensional datasets is a difficult analytic task, made possible through the use of well-suited methods. Independent Component Analysis (ICA) (Beckmann et al., 2005) has become a standard approach in this field, due to its power at detecting and disentangling overlapping, but statistically distinct, signals from noisy, high-dimensional data. The traditional application of ICA and other RSN analyses have focused on static analysis of the images, implicitly assuming that the networks are unaltered throughout the duration of each resting-state scan (Van Den Heuvel and Pol, 2010). The assumption that brain states are static for many minutes has the effect of averaging over the spatial and temporal variability of networks (Hutchison et al., 2013b).

Although the spatial structure of these RSN patterns is similar throughout the population, their exact structure and dynamics in time vary considerably between individuals (Song et al., 2008; van den Heuvel et al., 2009; Adelstein et al., 2011; Wei et al., 2011). Further, even within individuals, the modes reconfigure dynamically on timescales ranging from seconds to years (Honey et al., 2009; Shehzad et al., 2009; Chang and Glover, 2010; Meindl et al., 2010; Van Dijk et al., 2010; Bassett et al., 2011). Differences in an individual's RSN dynamics are important in part because they may serve as useful biomarkers for a variety of neurological dysfunctions, including schizophrenia (Damaraju et al., 2014; Ma et al., 2014; Rashid et al., 2014, 2016; Miller et al., 2016), bipolar disorder (Rashid et al., 2014, 2016), autism (Falahpour et al., 2016; de Lacy et al., 2017), depression (Demirtaş et al., 2016), post-traumatic stress disorder (Li et al., 2014), attention deficit/hyperactivity disorder (Ou et al., 2014), and mild cognitive impairment (Chen et al., 2016). For example, multiple studies have shown that in schizophrenia, regions of default mode, auditory, motor, and visual resting state networks show differences in their correlations, when compared to a control population (Garrity et al., 2007; Jafri et al., 2008; Woodward et al., 2011). Patients with schizophrenia also show abnormalities in dynamic dwell times in large-scale RSNs (Damaraju et al., 2014).

Technological innovations in brain imaging such as SENSitivity Encoding (SENSE) and simultaneous multi-slice (SMS) acquisitions now allow for improved temporal resolution, enabling investigation of the true temporal dynamics of brain function (Pruessmann et al., 1999; Feinberg and Setsompop, 2013; Barth et al., 2016). One emerging challenge with the advent of the fast acquisition of brain dynamics is the appropriate choice of methods to analyze and interpret data. It follows that much intense work has gone beyond static analyses, calculating RSN structure and activation as they vary in time to investigate a variety of measures of RSN dynamics. Many of these time-resolved RSN analyses use a windowed version of a traditional method, such as correlation (Chang and Glover, 2010; Sakouglu et al., 2010; Handwerker et al., 2012; Jones et al., 2012; Hutchison et al., 2013b; Allen et al., 2014; Preti and de Ville, 2017) or ICA (Beckmann and Smith, 2005; Hutchison et al., 2013a). Windows are then shifted in time and the measures recalculated, leading to methods for characterizing dynamic connectivity (Calhoun et al., 2014; Calhoun and Adali, 2016; Keilholz et al., 2017; Preti and de Ville, 2017). These most common RSN extraction methods make no explicit assumptions about the intrinsic temporal structure of the fMRI data (Kiviniemi et al., 2011; Calhoun and de Lacy, 2017). We note that there are alternatives to the windowed methods such as the wavelet coherence transform approach, which performs a time-frequency decomposition of the resting state signals from a pair of voxels or regions of interest (Chang and Glover, 2010). Another non-windowed approach is to fit a hidden Markov model, which uses Bayesian inference to infer states and their dynamics with the assumption that the system is in precisely one state at any given time (Vidaurre et al., 2017).

In this work, we present a new framework based on dynamic mode decomposition (DMD) for analyzing resting state BOLD fMRI data. DMD is a spatiotemporal modal decomposition technique (Brunton et al., 2016; Kutz et al., 2016a) ideally suited to extract coherent modes from rs-fMRI data. Similar to ICA, DMD decomposes a signal into constituent coupled spatiotemporal modes (Rowley et al., 2009; Schmid, 2010; Tu et al., 2013). Unlike ICA, DMD constrains the modes to be temporally coherent; specifically, each mode oscillates at a fixed frequency while exponentially growing or decaying. This temporal coherence constraint produces more robust estimates of spatial modes by leveraging the assumption that RSNs have coherent dynamics within short windows of time, while automatically allowing for any phase differences between regions of a network. Thus, in addition to a spatial map of activation, DMD also estimates the temporal frequencies associated with each spatial mode.

**Figure 1:**
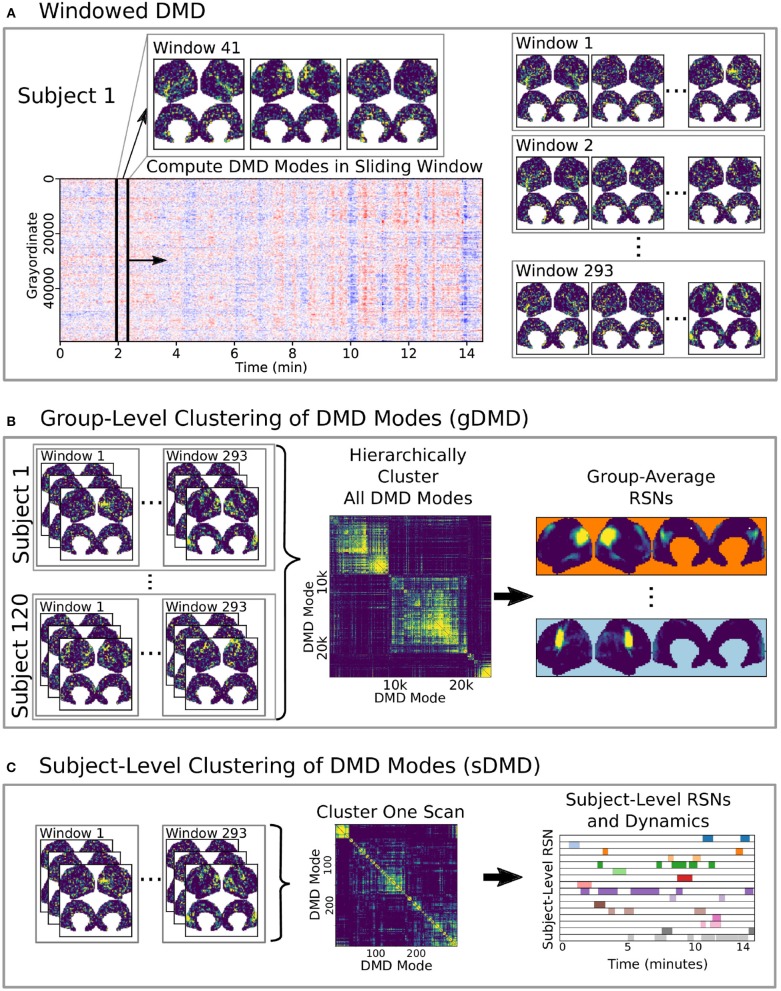
A schematic of our method using DMD to analyze resting state BOLD fMRI data. **(A)** Spatiotemporal DMD modes are extracted from short sliding windows of rs-fMRI data from 120 subjects from the Human Connectome Project. We use 23 s windows and slide in 3 s steps over each 15 min scan. **(B)** Group-DMD (gDMD) clusters the full set of modes from all 120 subjects to extract group-averaged RSNs. **(C)** Subject-level DMD (sDMD) clusters only the subset of modes from a single subject, which yields both individualized RSNs and their corresponding dynamics.

**Figure 2:**
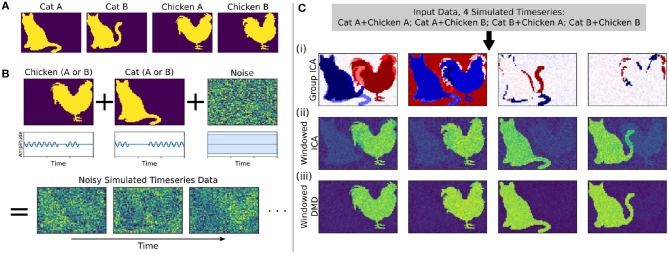
In a synthetic dataset with two “cat” modes and two “chicken” modes, windowed DMD performs better than group ICA and windowed ICA to extract the true underlying modes. **(A)** The synthetic cat and chicken modes. **(B)** The simulated timeseries data comprises intermittently pulsing cat/chicken modes plus white noise. **(C)** A comparison of the modes extracted from the simulated data using group ICA, sliding window ICA, and sliding window DMD. In (i), traditional group ICA does not separate the modes, which are neither fully spatially or temporally independent. In (ii), ICA modes computed in sliding windows and averaged over windows distinguishes each mode, but the results are noisy and contains shadows from the other modes. In (iii), DMD modes computed over sliding windows yield modes that are significantly closer to the true modes. In (ii) and (iii), the absolute value of each mode was taken before averaging, so that the colormap is different from that of (i).

**Figure 3:**
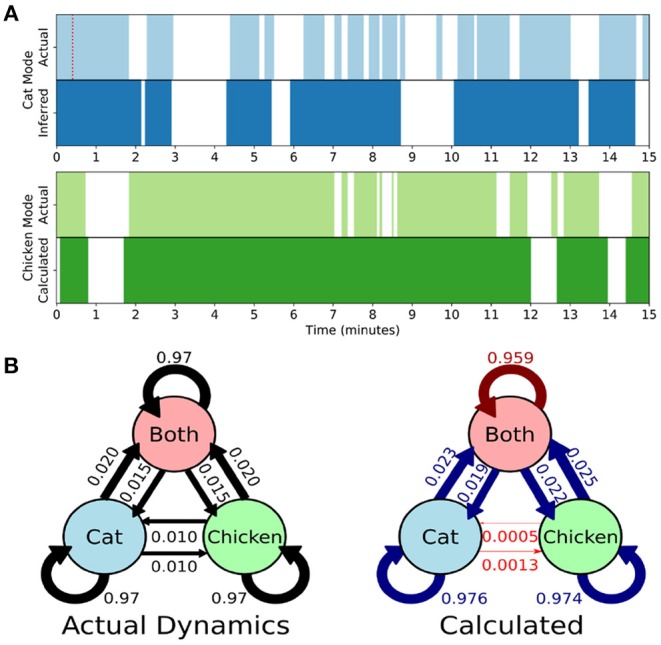
Single-subject DMD (sDMD) characterizes single-trial dynamics in a synthetic dataset by identifying the time windows during which each mode is present. **(A)** The actual (top) and calculated (bottom) dynamics for the cat mode (blue) and chicken mode (green). The red dotted line on the top panel indicates the length of the sliding window. **(B)** The true state dynamics are governed by a Markov network (left), and sDMD infers an approximation of these dynamics (right). The inferred transition probabilities are close to the true weights (over-estimates are in blue, and under-estimates are in red). Note, however, that these probabilities must be interpreted carefully: the smoothing of dynamics over the time-scale of the sliding windows means that rapid cat↔chicken transitions within a single window are extracted as “Both,” so that these transitions are under-estimated.

Here we develop and validate a novel method to extract reproducible, time-resolved RSNs based on DMD. DMD is computed within a short sliding window of data, and the spatial components of the DMD, known as DMD modes, represent coherent spatial correlations (Figure 1A). We first consider group-DMD (gDMD), where hierarchical clustering discovers stereotypical modes present within the full population (Figure 1B). We also apply DMD independently to single scans (sDMD), as illustrated in Figure 1C, which allows us to extract subject-level modes and their corresponding dynamics. To validate this method, we consider a synthetic dataset consisting of two dynamic, intermittently active, partially overlapping modes. gDMD outperforms both traditional and windowed ICA at extracting these modes from noisy data (Figure 2), and sDMD extracts the correct temporal dynamics to a temporal resolution matching the length of the sliding window (Figure 3).

We then analyze rs-fMRI data from 120 individuals in the Human Connectome Project (HCP) dataset (Glasser et al., 2013; Smith et al., 2013; Van Essen et al., 2013). The gDMD modes of the rs-fMRI data are strongly clustered, with clusters and sub-clusters corresponding directly to canonical RSNs. On a single-subject level, sDMD extracts and characterizes individualized RSN modes and their dynamics at a resolution of ~3 s. To assess our time-resolved results, we compute the subject-level default mode networks (DMNs) through DMD and find that the individualized variations we observe agree with those derived through group-ICA (gICA) and dual regression. Importantly, this agreement is in spite of the fact that our DMD approach uses only data from a single subject, whereas the gICA approach regresses against a reference RSN computed from the entire population. We show that the sDMD modes resemble the gICA modes more closely and more robustly than subject-level DMNs computed via single-scan spatial ICA. Our subject-level analysis lends well to characterizing the RSN dynamics, including how frequently each RSN is active and the probabilities of transitions between different RSNs. We show that the sDMD derived RSN occupancies are reproducible within each individual across independent scans, and these dynamical properties may be used to build subject-level network representations of RSN dynamics. These results lay the groundwork for the application of the DMD family of methods to fMRI data; DMD is a modular approach that extends easily to multi-resolution analyses and control theoretic frameworks, a key advantage over ICA-based methods that have no directly analogous extensions. We suggest that DMD is an improved method for robust, reproducible extraction of functional connectivity modes from short windows of rs-fMRI data, and these time-resolved modes are ideally suited to characterize RSN dynamics on the single-subject level.

## 2. Results

Here we present results using our method combining dynamic mode decomposition (DMD) and unsupervised clustering to extract resting state networks (RSNs) from our synthetic dataset and the Human Connectome Project (HCP) data. We show results at both the group level (gDMD) and the subject level (sDMD), as summarized in Figure 1. The sDMD results extract reproducible, time-resolved RSNs, and their transition dynamics for individual scans. Details of our approach are described in the section Methods. Code to assist in downloading the correct data, run all analyses described in this paper, and generate the corresponding figures are openly available at https://github.com/kunert/DMD_RSN.

### 2.1. Synthetic Data

We first consider a synthetic dataset containing “cat” modes and “chicken” modes, shown in Figure 2A. Each mode has two variants, and we conduct a total of four simulations corresponding to the different combinations of Cat A/B and Chicken A/B. In each simulation, the chicken mode and cat mode are each intermittently pulsing in the presence of a high level of white noise, as depicted in Figure 2B.

We use three methods to extract the underlying modes from these noisy simulations, with the results shown in Figure 2C. We first consider Group ICA, which concatenates the four simulations into a single dataset from which the ICA modes are computed. In this synthetic case wherein the modes are neither fully spatially nor temporally independent, this approach lumps the underlying patterns together and fails to separate them spatiotemporally.

To resolve modes in time, we instead utilize a windowed approach: we compute ICA/DMD modes from short sliding windows of data within each individual scan. Specifically, we compute modes within 32-frame windows sliding by 4 frames, producing a total over 293 overlapping windows per each 1,200-frame scan. We average together subsets of the resultant modes to calculate the optimal reconstruction of the original underlying patterns. The ICA/DMD approaches shown here are identical except for the one line of code in which each technique is used to extract modes from a window of data. In addition to DMD mode extraction being considerably faster than ICA [taking (1.7±0.1) s per scan for windowed DMD and (7.0±1.0) s per scan for windowed ICA], Figure 2C shows that windowed DMD modes yields a cleaner result than windowed ICA, the results of which are noisier and contain shadows from other modes. This windowed DMD technique forms the basis of both our sDMD and gDMD approaches.

Rather than correlating modes against a known pattern, the gDMD/sDMD approaches use unsupervised clustering to group together similar modes from different windows. This yields the time dynamics of each mode, since we know the time windows in which each mode does or does not appear. In this synthetic data, we can compare these inferred time dynamics against the ground truth, as in Figure 3A. The inferred dynamics match the ground truth, though they are smoothed over the time-scale of the window such that more rapid transitions are not captured. We can further use these inferred timeseries to estimate the transitions between states of the simulation, as in Figure 3B. The true state dynamics are governed by a Markov network which switches between only the cat mode being active, only the chicken mode being active, and both modes being active simultaneously. The window-smoothing effect results in an undercounting of rapid cat↔chicken transitions, which further highlights that the resolution of the inferred dynamics is limited by the window size; otherwise, the inferred transition probabilities are close to the underlying ground truth.

### 2.2. rs-fMRI Data: Extracted Group-Level Modes Resemble Resting State Networks (RSNs)

The BOLD data we analyze is a collection of 15-min resting-state scans from 120 unrelated individuals from the HCP. Each scan has 1,200 frames acquired at a temporal resolution of ~0.72 s. The data had been pre-processed with the HCP minimal pre-processing pipeline Glasser et al. (2013) and is freely available to download at https://www.humanconnectome.org/. As part of this pre-processing, the data are mapped to the “grayordinate” space, where each point maps onto the cortical surface.

**Figure 4:**
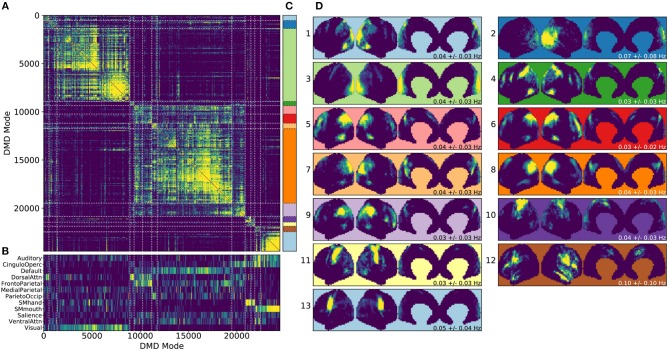
Results from gDMD using modes extracted from scans of 120 individuals, revealing clusters closely resembling known RSNs. **(A)** The hierarchically clustered correlation matrix between gDMD modes. **(B)** Overlap of each mode with RSNs from Gordon et al. (2016). **(C)** Clusters automatically extracted from the hierarchical clustering results. A different threshold can be chosen to derive finer or coarser sub-clusters. **(D)** Averaged modes within each of the 13 clusters, with the corresponding DMD temporal frequencies. Several of the automatically identified DMD mode clusters visually correspond to canonical RSNs.

As we did for the synthetic data, we apply DMD to short sliding windows within each individual scan In the results shown here (Figure 4), we use windows of 32 frames sliding by 4 frames, producing a total over 293 overlapping windows per each 1,200-frame scan. In time, this corresponds to ~23 s windows sliding in ~3 s increments. We truncate to the first 8 modes from each window. Our results presented here are relatively robust to the number of modes calculated within each window, as described further in Appendix C (Supplementary Material).

To show that the DMD modes thus computed resemble known RSNs, we use an unsupervised clustering approach to automatically identify and label clusters of modes, then compare them to canonical RSNs. Specifically, we perform agglomerative clustering to hierarchically cluster our set of DMD modes based on the average spatial correlation between clusters (Bar-Joseph et al., 2001; Mullner, 2011) (as implemented in the cluster.hierarchy package of SciPy 1.0.0; Jones et al., 2001). In gDMD, we consider as a group all the DMD modes from all windows of 120 individuals in the dataset. The hierarchically clustered correlation matrix is shown in Figure 4A, where the modes have been reordered so that this matrix is strongly block diagonal. In other words, groups of modes cluster naturally. Figure 4B shows the correlation of each DMD mode to each of a set of reference RSNs (Gordon et al., 2016). Importantly, the automatically-grouped clusters appear to correspond to distinct RSNs. For instance, the strongly clustered block in the lower-right of the correlation matrix shows very strong correlations to the mouth sensorimotor RSN.

The clustering establishes boundaries that separate modes into distinct clusters, shown in different colors in Figure 4C. This procedure uses a threshold that determines the resultant size and granularity of the clusters. Here we choose a threshold on the cophenetic distance (i.e., the “distance” threshold option of the scipy.cluster.hierarchy function fclust; Jones et al., 2001) of 0.955, a relatively coarse grouping of the modes into 13 clusters whose averages are shown in Figure 4D. These cluster averages, extracted at the population level, appear to correctly resemble known resting state networks. For instance, cluster 3 (light green) resembles the visual network, cluster 8 (dark orange) resembles the default network, and cluster 10 (dark purple) resembles the sensorimotor hand network.

Every DMD spatial mode has a corresponding temporal eigenvalue, which gives information about the oscillation frequency of that mode in time. Although these eigenvalues have not been used in the clustering process, they describe the characteristic oscillation frequency of each cluster. The mean and standard deviation for each cluster's oscillation frequency is shown in Figure 4D. As a validation that RSNs have low frequencies of oscillation, these frequencies are generally below or around 0.10 Hz. Further analysis of the frequency content of extracted gDMD clusters is discussed in Appendix B (Supplementary Material).

### 2.3. Subject-Level Modes and Dynamics

**Figure 5:**
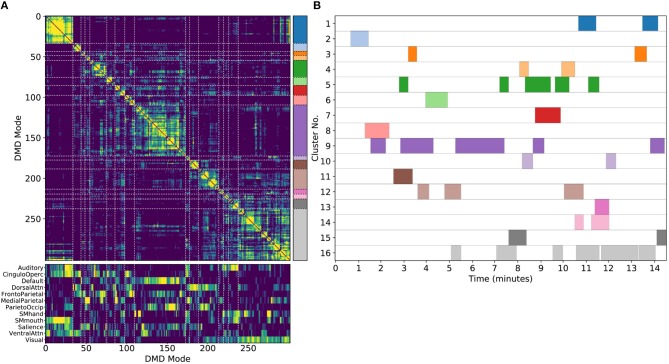
Results from sDMD using modes extracted from a single scan. **(A)** The hierarchically clustered correlation matrix between the sDMD modes, and their overlap with the RSNs of Gordon et al. (2016). **(B)** Temporal activation of each cluster within the scan. Each row corresponds to a distinct cluster, and columns are colored in if the corresponding cluster is active within that time window.

One key advantage of our approach using DMD followed by unsupervised clustering is that it can be performed equally well on any subset of modes. We are particularly interested in performing the analysis on single scans, which we refer to as sDMD. Figure 5 shows the results from hierarchically clustering modes from a single 15 min scan. Similarly to the group results, this similarity matrix is strongly block diagonal, and the blocks correspond to different canonical RSNs (Figure 5A, bottom, correlates these modes with the parcellation of Gordon et al., 2016). Note that we use a slightly lower threshold on the cophenetic distance than in Figure 4 (0.95 instead of 0.955), yielding a larger number of finer-grained subclusters.

Importantly, in this single-scan analysis, the clusters are easily interpreted temporally: each cluster shows temporal dynamics defined by the time windows in which its constituent modes are found, as shown in Figure 5B. Largely, clusters are active over periods spanning many consecutive windows and extended periods of time. Notably, many different clusters are observed to co-occur in time. This overlap of modes in the same window poses no problems for the sDMD approach, but it violates the assumptions made by other time-resolved methods (such as hidden Markov models) that require the system is in a single state at any particular time.

### 2.4. Subject-Level Modes Capture Spatial Heterogeneity

Clusters derived by sDMD are able to reliably capture individual variability of RSNs. To quantify this feature, we compare one particular resting state network, the default mode network (DMN), as extracted by three different methods: sDMD, ICA, and group-ICA with dual regression (gICA). The gICA networks were computed by the HCP and are available on ConnectomeDB as part of the *High-level rfMRI Connectivity Analyses* data release. In short, this approach generates high-quality group ICA modes using the dataset of 1,200 individuals then uses dual regression to adapt each group mode to the heterogenous structure seen within a particular scan.

**Figure 6:**
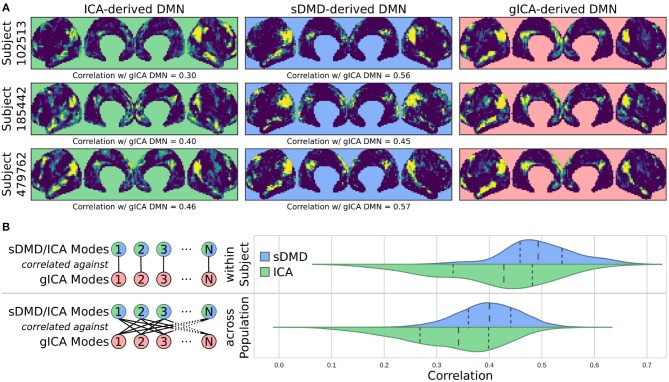
sDMD is able to capture the individual heterogeneity in spatial DMN structure. **(A)** Each column shows examples of subject-level DMNs as calculated by: (i) traditional ICA, (ii) sDMD, and (iii) group-ICA with dual regression (gICA). gICA yields the cleanest modes but requires a high-quality group-level mask, whereas ICA and sDMD use only data from a single scan. **(B)** The spatial correlation of ICA/sDMD modes with gICA modes. We compare how strongly each ICA/sDMD mode correlates with the gICA mode from the same individual, as well as how strongly it correlates with the full population's gICA modes. Both methods find modes which correlate more strongly with DMNs from the same individual, indicating that they all capture similar subject-level variations in DMN structure. sDMD outperforms ICA in this regard, however, and correlates more strongly with the gICA modes. At the same time, sDMD provides more unambiguous temporal information than either approach.

Figure 6 compares the DMNs extracted by ICA, sDMD, and gICA. Specifically, we use spatial-ICA on the entire window of scan data, as opposed to the sliding-window approach of sDMD. ICA and sDMD were both run multiple times with varying hyper-parameters, and for each method we choose the output that most strongly correlates with the canonical DMN (i.e., the DMN from Gordon et al., 2016). Specifically, ICA was performed multiple times as the fitting process is inherently stochastic, and will sometimes fail to extract a quality mode; thus we performed ICA on each scan ten times, each time randomly varying the number of modes in the range 8–16, and kept the mode which most strongly resembled the gICA DMN. sDMD, on the other hand, is inherently deterministic and was only performed once to produce a single set of modes; clustering of these modes was performed over a range of clustering parameters (the pre-clustering mode mask threshold, and the cophenetic distance threshold for forming clusters) and we kept the result most closely resembling the gICA mode. Importantly, the ICA and sDMD analyses were computed on data from single scans, whereas gICA produces personalized DMNs for each scan based on a population-derived DMN using dual regression. As shown in the examples from 3 different subjects in Figure 6A, qualitatively speaking, ICA and sDMD are both able to extract DMNs that resemble the personalized gICA DMNs. In particular, note that some of the spatial heterogeneity of DMNs for individual subjects is recapitulated in the ICA and sDMD networks.

We show that sDMD reliably outperforms ICA in quantitative comparisons with gICA on a subject-by-subject basis. Figure 6B shows that DMNs from both ICA and sDMD correlate significantly more strongly with gICA results from the same individual (bottom panel) than with gICA results from the rest of the population (top panel). Specifically, the distribution of correlations for ICA has a lower mean and longer tail of low correlation values than the distribution of correlations for sDMD. This result indicates that sDMD extracts similar individualized DMN structures as gICA using data from only a single subject. Further, the sDMD analysis provides unambiguous time dynamics of the subject-level modes. The gICA or ICA modes can be correlated against the scan data to yield a measure of activation in time, but this is a continuous measure which requires thresholding and will be significantly nonzero in the case of any overlapping signal or noise. Our clustering approach, on the other hand, unambiguously identifies a cluster as active/inactive within a particular window of time.

### 2.5. Dynamic Properties of Modes Are Reproducible

Our proposed gDMD and sDMD analyses produce reproducible dynamics in addition to reliable spatial structures. This reproducibility criterion is critical for interpreting dynamic properties as meaningful reflections of the underlying functional networks. Useful dynamic properties to compute include how often each cluster is active, and how often pairs of clusters are active together (either simultaneously, or with one cluster transitioning into another). To assess reproducibility, we take advantage of the HCP resting state dataset, where each of the 120 subjects underwent four (4) different 15 min resting state scans. Specifically, we repeat the same analysis for each of these four sets of scans separately and quantify to what extent our DMD analysis extracts clusters with similar averaged modes and dynamic properties. Here we examine dynamic networks extracted by gDMD, looking at two simple measure of dynamics: the occupancy of and the transfer matrices between clusters. Next, we quantify the reproducibility of these dynamics at a single-subject level by using sDMD on each of the 4 scans for each subject.

**Figure 7:**
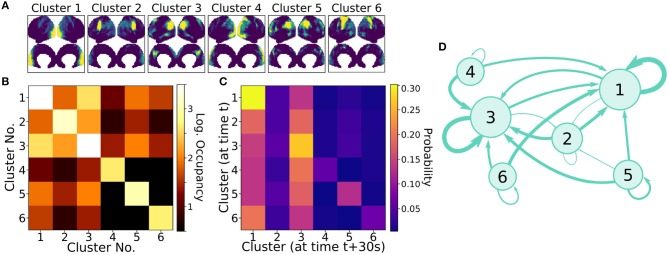
Dynamic properties of networks as extracted by gDMD revealed by the occupancy and transitions of clusters. **(A)** gDMD was performed on four independent sets of scans of the same population of 120 individuals. The six clusters plotted here are those extracted most reliably from the four datasets. **(B)** The occupancy matrix *O*_*ij*_: each entry shows how often cluster *i* and *j* are active simultaneously (the diagonal simply shows how often cluster *i* is active). **(C)** The transfer matrix *T*_*ij*_: this shows the probability that if cluster *i* is active, cluster *j* will be active 30 s later. **(D)** The transfer matrix can be interpreted as a network which characterizes RSN dynamics.

Clusters were paired between the four independent scans based upon the spatial similarity of their averaged modes; this matching procedure, as well as visualizations of all extracted average modes, is detailed in Appendix A (Supplementary Material). Figure 7A shows the six most highly correlated clusters. Figure 7B shows the occupancy matrix *O*_*ij*_ of these 6 clusters, where the value in the *i*th row and *j*th column corresponds to how often clusters *i* and *j* are simultaneously active. The diagonal *O*_*ii*_ shows how often each cluster *i* is active, and the matrix is symmetric. Next, Figure 7C shows a transfer matrix *T*_*ij*_, which contains the probability that, if cluster *i* is active, then cluster *j* will be active 30 s later. This matrix not symmetric, and it encodes rich dynamical information that can be interpreted as a dynamic network model, as shown schematically in Figure 7D.

**Figure 8:**
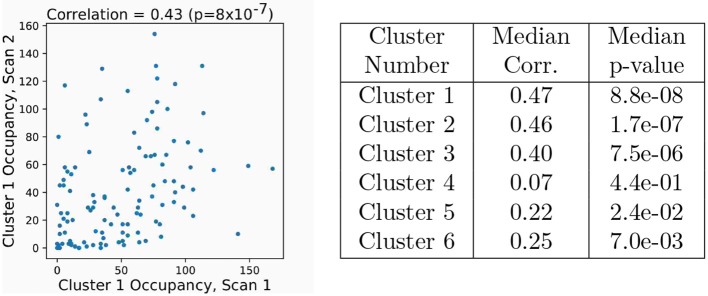
Occupancy of clusters computed by sDMD is correlated across different scans of the same subjects. The six clusters are the same as in those in Figure 7. Spearman's rank correlation is computed of occupancies between all pairs of the four scans. The plot at left shows the occupancy of Cluster 1 (*O*_11_) for each subject in two different scans (REST1_LR and REST2_LR). There is a significant correlation; subjects with high activity in one scan are more likely to have high activity in another. In the table at right, we have correlated all possible pairs of the four scans and report the median correlation (and corresponding *p*-value). The correlations have *p* < 0.05 for all but mode 4.

We next use these same 6 cluster from the gDMD and repeat the analysis for individual single scans, analyzing the extent to which the dynamic properties are unique to each individual. The occupancy of each cluster was broken up into how many of its modes are from each individual scan, and Figure 8 shows the results of the subject-level occupancy correlation between two different sets of scans of the same individuals. Correlation coefficients and *p*-values are calculated for all possible pairs of the 4 different scans, and Figure 8 reports the median correlation across all scan comparisons and the corresponding *p*-value. For five of the six clusters, occupancy results from different scans of the same individual are positively correlated, with the strongest and most significant correlations observed for the most active clusters. Note that the high *p*-value of Cluster 4 does not imply that it is not meaningful, but rather suggest that its activity may not correlate within an individual across different scans (as would be the case, for example, if the activity of that RSN was uniformly probable across the population). Thus, our approach extracts reproducible, individualized dynamic properties of resting state networks.

## 3. Discussion

In this work we present a novel framework based on the dynamic mode decomposition (DMD) for extracting time-resolved resting state networks at both the group (gDMD) and single-subject (sDMD) levels. DMD decomposes high-dimensional time-series data into a sum of dominant coupled spatiotemporal modes. One way to think about DMD is that it is similar to a combination of applying principal component analysis (PCA) in space and the Fourier transform in time, as it extracts both spatial maps and their coupled frequency content simultaneously. We use DMD to extract coherent spatiotemporal modes from short, sliding windows of data, and underlying patterns are revealed by unsupervised clustering. We validate this approach on a synthetic dataset, in which we find that this windowed DMD approach separates the underlying spatiotemporal modes more cleanly than windowed ICA, and that it correctly infers the underlying dynamics down to the time-scale of the sliding window length. We then demonstrate the application of gDMD and sDMD to rs-fMRI using data from 120 subjects from the Human Connectome Project (HCP). The gDMD clusters correspond to the average modes within a population and closely resemble canonical RSNs. The sDMD use only data from a single scan, calculating subject-level RSNs and their temporal dynamics simultaneously. When sDMD is used to extract subject-level Default Mode Networks from the data of a single scan, it does so more reliably than ICA, while also having unambiguous time resolution. Finally, we show that the extracted temporal dynamics are highly reproducible within subjects and between trials.

DMD extracts additional information beyond what we have fully considered within the scope of this work. By construction, each DMD has an intrinsic temporal frequency. We have made note of these frequencies (e.g., as labels within Figure 4) but have drawn no conclusions from them beyond noting that they are plausible compared to the known literature (i.e., generally <0.1 Hz). However, the preliminary analyses in Appendix B (Supplementary Material) suggest that networks may have distinct and reproducible frequency content which could be characterized by future studies.

Another aspect of the DMD modes which we have not explored fully here is the phase information in each mode and how it relates to resting state network organization. The phase information is particularly interesting given that DMD modes are allowed to spatially overlap. Future studies should consider if there is a systematic difference in the phases of overlapping regions, and whether this is related to the RSN dynamics (e.g., the network in Figure 7). If the activation of the overlapping region was phase-lagged behind the rest of the mode, and if this was followed by the activation of the overlapping RSN, then these overlaps could potentially be interpretable as mediating regions the transition between the modes.

Beyond data that has already been pre-processed and mapped onto “grayordinate” cortical surface space, it may be desirable to analyze volumetric data directly. In principle, there is no reason why our DMD approach cannot be applied equally well to voxel data; nevertheless, the quality of the networks thus extracted would be unknown and must be thoroughly characterized. Similarly, care should be taken when applying our approach to data acquired at different spatial and temporal resolutions. We have made all of our code openly available in the hope that these analyses can be performed readily and reproducibly.

This demonstration that DMD is capable of extracting modes reliably from short windows of data, with similar or better performance than ICA, lays the groundwork for future methodological developments. Indeed, a key motivation for the use of DMD is that a number of powerful extensions to DMD have been developed that could be readily applied to BOLD fMRI data. DMD with Control (DMDc [62]) uses a control theoretic framework, allowing intrinsic dynamics to be estimated from those driven by external stimuli. DMDc is a natural framework to analyze task-based fMRI scans, as it distinguishes intrinsically-driven dynamics (i.e., downstream excitement of modes by other modes) from externally-driven dynamics (i.e., those driven by the external task). Rather than the simple correlations captured by the networks extracted in the current paper, this future development should allow for the extraction of networks which capture the causal relationships between RSNs. Another approach, multi-resolution DMD (mrDMD; Kutz et al., 2016b) improves the quality of the temporal information extracted over multiple timescales. These straightforward extensions of DMD to multi-resolution and control frameworks have no direct counterpart in ICA, giving DMD a critical advantage over ICA in future analyses of multi-scale signal extraction and control. Similarly, Optimized DMD (Askham and Kutz, 2017) is an implementation of the DMD algorithm which is more computationally expensive than Exact DMD (the algorithm we use here), but significantly improves the precision of the extracted frequency information, which would enable the study of how characteristic frequencies may vary between networks, as suggested by the preliminary analyses of Appendix B (Supplementary Material).

It is increasingly clear that RSN dynamics are significantly altered by neurological disorders, and their impact on the spatial and temporal structures of RSNs may be diverse and highly individualized. Rather than searching only for patterns in RSNs strongly resembling those seen in the general population, it may be fruitful to explore methods that are agnostic to the average. We propose that DMD is an approach well suited for data-driven extraction of individualized structure and dynamics of networks from single scans, and we believe the development of the DMD family of methods opens new doors for exploring and characterizing whole-brain dynamics as captured by fMRI.

## 4. Methods

### 4.1. Human Connectome Project rs-fMRI Data

We used rs-fMRI data from 120 unrelated individuals in the Human Connectome Project (HCP) dataset as provided by the WU-Minn Consortium (Smith et al., 2013; Van Essen et al., 2013). The HCP acquired four separate resting state scans using a multiband pulse sequence (multiband factor = 8, FA = 52°), each scan having 1,200 time points (14.4 min, TR: 720 ms, TE: 33 ms, FOV: 208 × 180 mm, Matrix: 104 × 90, with 72 slices; Glasser et al., 2013. For each scan, an equal number of volumes with left-right and right-left phase encoding directions were acquired. To correct the fMRI scans for gradient distortions, the HCP also acquired two spin echo EPI images with reversed phase encoding directions, with a unique pair of spin echo EPI images for each of the resting state acquisitions (Sotiropoulos et al., 2013). These spin echo EPI images were then used to accurately spatially normalize the fMRI volumes to the structural scans. For the remainder of this document, we label and refer to the four resting-state runs as “REST1_LR,” “REST1_RL,” “REST2_LR,” and “REST2_RL”.

The resting state scans had already been preprocessed by the HCP Consortium using the HCP minimal preprocessing pipeline (Glasser et al., 2013). In addition to gradient distortion correction, this preprocessing includes fMRI denoising using FIX (Salimi-Khorshidi et al., 2013), masking, motion correction, registration and interpolation of the timeseries onto the cortical surface (the CIFTI “grayordinate” space). Included in this preprocessing was the application of FreeSurfer to generate mesh representations of the cortical surface. These FreeSurfer surface meshes were upsampled to ~164 k vertices, and subsequently downsampled to ~32 k vertices, resulting in an average vertex spacing of roughly 2 mm at the cortical midthickness level. The resting state time series were then mapped onto this low-resolution 32 k vertex mesh. This surface-mapped data consists of 91,282 “grayordinates” (~60 k surface points mapping the cortical surfaces, along with ~30 k subcortical voxels). In this manuscript, we restrict our attention to the 2*D* cortical surface mesh. Note, however, that DMD could be similarly applied to 3*D* voxelized data (see section Discussion).

All data used in this manuscript are freely available for download from the HCP. Additionally, scripts for downloading the correct data, running all analyses described within this paper, and figure generation are openly available at https://github.com/kunert/DMD_RSN.

### 4.2. Synthetic Data Simulations

#### 4.2.1. Synthetic Data Modes

The synthetic data *X*_*ij*_ at time *t*_*k*_ consists of a sum of the intermittently pulsing images of cat *i* and chicken *k* with noise:

(1) $$X_{ij}(t_{k})=a_{c}\sin(2\pi f_{c}t+\phi_{c})\cdot C_{i}+a_{k}\sin(2\pi f_{k}t+\phi_{k})\cdot K_{j}+N$$

where *C*_*i*_ ∈ {*C*_*A*_, *C*_*B*_}/*K*_*i*_ ∈ {*K*_*A*_, *K*_*B*_} are the 75 × 50 pixel cat/chicken images. Amplitudes *a*_*c*_ and *a*_*k*_ are 1 or 0 depending on the system state. Each mode has a frequency *f*_*c*_, *f*_*k*_ and phase ϕ_*c*_, ϕ_*k*_. These results use *f*_*c*_ = 0.045 and *f*_*k*_ = 0.071 cycles per timestep (if δ*t* ≈ 0.73 s, as for rs-fMRI data, these are about 0.06 and 0.1 Hz, within the appropriate range for RSNs) and ϕ_*c*_ = 1.6 rad and ϕ_*k*_ = 0.7 rad. *N* is a 75 × 50 matrix of uniform random noise with amplitude 5.

Each simulation consists of 1,200 timesteps, chosen to match the length of an HCP scan. The state of the system is governed by an underlying Markov network, with the possible states being (*a*_*c*_, *a*_*k*_) ∈ {(1, 0), (0, 1), (1, 1)} (i.e., just the cat, just the chicken, or both). The transition probabilities at each timestep are shown in Figure 3B.

Windowed ICA, windowed DMD, and sDMD were calculated identically to how they were calculated for the rs-fMRI data, which is discussed in detail throughout the rest of the Methods. The only computation unique to the synthetic dataset took place in the mode reconstruction procedure, which yielded the results in Figure 2C (ii) and (iii). In principle, we could have applied the mode clustering procedure of gDMD to both methods, but this would yield a potentially ambiguous comparison for two reasons: first, it would not clarify whether the DMD modes were intrinsically cleaner, or if they were simply better suited to this particular clustering procedure; second, it would require an intensive parameter optimization to ensure that both methods were returning their optimal results. We instead use the following procedure:

1. Calculate the DMD modes (or ICA modes) from every window: *M* = {*M*_1_, *M*_2_, …}. To allow for the averaging of modes, take their absolute value.
2. Correlate every mode in *M* against a ground-truth mode *C* (i.e., *C* is one of the cat/chicken modes).
3. Re-order *M* in descending order of the magnitude of the correlation with *C* (i.e., *M*_1_ is now the mode which most strongly resembles *C*, *M*_2_ is the second strongest, etc.)
4. Average together the first *k* modes, choosing *k* to maximize the average mode's correlation with *C*.

Clearly, this is a supervised method which requires correlation against a reference mode, rather than the unsupervised approach we take when clustering the rs-fMRI results. However, it provides an upper bound on the quality of the averaged mode which clustering could return. It is thus representative of the best possible outcome of using the gDMD approach using either ICA or DMD to calculate the modes in each window, thereby establishing that DMD does a better job at extracting spatiotemporally decoupled modes from the short windows of noisy data considered here.

#### 4.2.2. Synthetic Data Dynamics

To assess whether the inferred dynamics are reasonable, we compared them to the known transition probabilities within the underlying Markov network. The reconstruction of the dynamic traces used the same analysis pipeline which we developed for analyzing single-subject rs-fMRI data, as detailed in sections 4.3, 4.5, and 4.7. In brief, we do the following:

1. Calculate the DMD modes within all sliding windows for a single subject.
2. Hierarchically cluster the modes, setting the clustering threshold so as to form two large clusters. In practice, these are seen to be the Cat and Chicken modes corresponding to that particular run. Modes within each clusters are correspondingly labeled as cat modes or chicken modes.
3. For each timepoint, tally how many of its corresponding windows contain a cat and/or chicken mode. This generates the dynamic traces as seen in Figure 3.

Note that since we are using overlapping sliding windows, each timepoint belongs within multiple windows; in Figure 3, the cat and/or chicken modes are considered to be active at a given timepoint if any of the corresponding windows contain a cat and/or chicken mode, which has the effect of smoothing the dynamics over the time-scale of the sliding window.

The reconstruction of the dynamics thus far is identical to our method for the fMRI data, but diverges on two key points when comparing the dynamics against the ground truth: first, the fMRI transition matrix (as in Figure 7) compares the state at a given time *t* against the state a full 30 s (or 40 timesteps) later. The synthetic data analysis, however, compares the state at a given time *t* to the state only one timestep later. This is necessary to compare against the underlying Markov probabilities. Second, the synthetic dataset is Markovian, with precisely one active state at each timestep, where “both” states being active is an explicit state of the system; the network in Figure 7D, however, does not have this constraint, and should not be interpreted as a Markov network.

### 4.3. Dynamic Mode Decomposition

This section describes the specific DMD algorithm implemented in this manuscript (Brunton et al., 2016; Kutz et al., 2016a). Each scan is broken into windows of data, and data from each window is collected into a data matrix **X**, where each row represents one of the *n* grayordinates and each column $\vec{x}$ is one of the *m* time snapshots in that particular window:

(2) $$X=\left[\begin{array}{cccc} | & | & \; & |\\ {\vec{x}}_{1} & {\vec{x}}_{2} & \ldots & {\vec{x}}_{m-1}\\ | & | & \; & | \end{array}\right].$$

Here ${\vec{x}}_{k}$ is a vector in grayordinates space giving the BOLD signal at time index *k*. Next, we also define the time-shifted data matrix **X′**, which is defined similarly but with each column advanced forward by a single timepoint:

(3) $$X^{\prime }=\left[\begin{array}{cccc} | & | & \; & |\\ {\vec{x}}_{2} & {\vec{x}}_{3} & \ldots & {\vec{x}}_{m}\\ | & | & \; & | \end{array}\right].$$

The goal of DMD is to describe the matrix **A** that best maps ${\vec{x}}_{k}$ onto ${\vec{x}}_{k+1}$, namely by solving for the eigenvalues and eigenvectors of **A**. In other words, we treat dynamics of the system as approximately linear and seek the **A** that best solves

(4) $$X^{\prime }=AX.$$

However, this **A** matrix is a *n* × *n* square matrix; for the HCP data, *n* = 91, 282 grayordinants, so **A** has approximately 10^10^ elements — this is a tremendously large number of unknowns, and they are poorly constrained by the limited data in **X** and **X′**. Further, we hypothesize that many of these brain areas have strong correlations, so their dynamics are relatively low rank and may be explained by far fewer modes.

The DMD algorithm takes advantage of the singular value spectrum (SVD) of the data matrix **X** to obtain the dominant eigendecomposition of **A** without actually computing **A**. We first take the singular value decomposition (SVD) of the data matrix X = UΣV^*^, which decomposes the **A** into the product of unitary matrices **U, V** and diagonal matrix **Σ**. Because the singular values and singular vectors are ordered by decreasing energy, this decomposition can be used truncated by taking the first *r* columns of **U** to form a *n* × *r* matrix **U_r_**. We may similarly form a *r* × *r* matrix **Σ_*r*_** and a *m* × *r* matrix **V_r_**.

This truncation gives the optimal rank-*r* reconstruction of the data matrix, $X\approx U_{r}\Sigma_{r}V_{r}^{*}$. The choice of the number of SVD modes *r* is equal to the resultant number of DMD modes *r* and is used as a parameter throughout this manuscript.

Taking the SVD allows us to then calculate the pseudoinverse $X^{†}=V_{r}\Sigma_{r}^{-1}U_{r}^{*}$ and simply solve for **A**:

(5) $$A\approx X^{\prime }X^{†}\approx X^{\prime }V_{r}\Sigma_{r}^{-1}U_{r}^{*}.$$

Given that the number of grayordinates is large, we do not consider the *n* × *n* **A** matrix directly, but instead project into the reduced-dimensional **U_r_** basis:

(6) $$\tilde{A}\equiv U_{r}^{*}AU_{r}=U_{r}^{*}X^{\prime }V\Sigma^{-1}.$$

Additionally, we scale each mode according to how strongly it is present in the original data, and each modes is scaled by the singular values **Σ_*r*_** as in Brunton et al. (2016):

(7) $$\hat{A}\equiv \Sigma^{-1/2}\tilde{A}\Sigma^{1/2}.$$

We then compute the eigendecomposition of this scaled, reduced dimensional $\hat{\text{A}}$ matrix:

(8) $$\hat{A}\hat{W}=\hat{W}\Lambda ,$$

where **Λ** is the diagonal matrix of eigenvalues λ_*j*_, and the columns of $\hat{\text{W}}$ are the eigenvectors of $\hat{\text{A}}$. These eigenvectors can be used to compute the dynamic modes:

(9) $$\Phi \equiv X^{\prime }V\Sigma^{-1/2}\hat{W},$$

where the *j*-th column of **Φ** is the dynamic mode ${\vec{\phi }}_{j}$. From the dynamic modes and corresponding eigenvalues, we can approximate the dynamics of the system as:

(10) $${\vec{x}}_{k}\approx \sum_{j=1}^{r}b_{j}\lambda_{j}^{k}{\vec{\phi }}_{j},$$

where the weights *b*_*j*_ can be fit as initial conditions for at *k* = 0. We can also write this in terms of a continuous time *t* by writing the dynamics in terms of a complex exponential:

(11) $$\vec{x}(t)\approx \sum_{j=1}^{r}b_{j}\exp\left((\gamma_{j}+i2\pi f_{j})\;\cdot \;t\right){\vec{\phi }}_{j}.$$

In the exponent, γ_*j*_ is the real-valued growth/decay constant, and *f*_*j*_ is the real-valued oscillation frequency of the mode in cycles per second (hertz). These are calculated from the real and imaginary components of the corresponding DMD eigenvalues:

(12) $$\gamma_{j}=\frac{\text{real}(\ln(\lambda_{j}))}{\Delta t},\;f_{j}=\frac{\text{imag}(\ln(\lambda_{j}))}{2\pi \;\Delta t},$$

where Δ*t* is time between measurements, in seconds. This change of units does not carry any additional information over the complex eigenvalue λ_*j*_, but γ_*j*_ and *f*_*j*_ are readily interpretable as standard growth/decay constants and oscillation frequencies.

Like the eigenvalues, the dynamic modes ${\vec{\phi }}_{j}$ can be complex valued. In other words, each element of ${\vec{\phi }}_{j}$ has both a magnitude and a phase. In this work, we consider only the magnitude of the dynamic mode $\left|{\vec{\phi }}_{j}\right|$; generally, when we refer to the “dynamic mode,” or when we do calculations such as spatial correlations, etc., we are referring to and using |**ϕ**_*j*_|. However, the complex-valued **ϕ**_*j*_ also contains the relative phase between regions, which is of potential interest for future analyses. For real-valued data ${\vec{x}}_{k}$, oscillatory modes appear as conjugate pairs $\left\{{\vec{\phi }}_{j},{\vec{\phi }}_{j^{\prime }}\right\}$ with the same spatial magnitudes $\left|\phi_{j}|=|\phi_{j^{\prime }}\right|$; thus the number of unique spatial patterns extracted from a data matrix will be ≤ *r*.

### 4.4. Independent Component Analysis

fMRI data is often processed using Independent Component Analysis (ICA) (Beckmann et al., 2005). Just as above, if we collect all data from a scan into a data matrix **X**, then we can formulate ICA as an attempt to solve the following:

(13) $$X^{T}=AS^{T}.$$

The goal of spatial ICA is to decompose the data into non-orthogonal, statistically independent source signals, which are the columns of the *n* × *r* matrix **S**. The *m* × *r* mixing matrix **A** gives the mixture of these signals, at each timepoint, which best approximates the original data.

However, because individuals subjects have their own unique time courses, comparing ICA components estimated at the subject-level is difficult, and consequentially, how to make inference about group-level components is not immediately clear. As an alternative, fMRI analyses often employ multi-subject ICA methods to estimate (1) population-based components, or (2) single-subject components informed at the group level. A variety of multi-subject methods have been developed, including temporal or spatial concatenation group ICA approaches (Calhoun et al., 2001; Lukic et al., 2001), higher-order tensor decomposition methods (Beckmann and Smith, 2005), and dual regression-based approaches (Beckmann et al., 2009; Filippini et al., 2009).

In this analysis, we use the FastICA algorithm as implemented in scikit-learn 0.18 (Pedregosa et al., 2011). FastICA attempts to maximize the statistical independence of the signals by maximizing the non-Gaussianity of the projected data, as detailed in Hyvärinen and Oja (2000). In order to directly compare DMD to ICA, we compare both algorithms at two spatial component levels: using components informed at the single-subject level and using components informed at the group level. For the single-subject-level comparison, we compute single-subject ICA components using sklearn's FastICA. For the group-level comparison, we use the group ICA and dual regression results generated previously by the HCP using Melodic's Incremental Group-PCA algorithm (MIGP) (Smith et al., 2014). MIGP is an iterative approach that incrementally incorporates time-series data for single subjects and updates a current running estimate of the set of spatial eigenvectors that best describes the time series data for the current set of incorporated subjects. MIGP generates a very close approximation to group-ICA components generated by classic temporal concatenation group-ICA approaches, but without the large computer memory requirements. The HCP then used dual regression to regress each individual subject's time-courses on the MIGP-generated group-ICA components.

In practice, DMD should prove faster than ICA for short windows of high-dimensional data. Consider a data matrix *X* with *m* variables and *n* timepoints, where we have *m* ≫ *n* (for example, the rs-fMRI data has *m* = 59, 412 grayordinates and windows of length *n* = 32). We compute *r* DMD modes in each window, where *m* ≫ *r* (our rs-fMRI analysis uses *r* = 8). Exact DMD consists of an SVD [with time-complexity $O\left(m^{2}n\right)$], two matrix multiplications [with $O\left(mnr+mr^{2}\right)$], and an eigendecomposition [with $O\left(r^{3}\right)$]. Since *m* ≫ *n, r*, the time-complexity is dominated by the term $O\left(m^{2}n\right)$.

By contrast, FastICA has a time-complexity of O(m(m+1)nx) Laparra et al. (2011), where *x* is the number of iterations required for convergence. In our case, this is approximately $O\left(m^{2}nx\right)$. In other words, the time-complexity of DMD scales similarly to a single iteration of FastICA, which can lead to considerable speed-up as FastICA can take many iterations to converge (by default, the scikit-learn implementation of FastICA will allow up to 200 iterations).

### 4.5. Sliding Window Mode Calculations

The rs-fMRI scans analyzed each consist of 1,200 individual timesteps, which we break up into sliding windows. We use a simple square window (as opposed to a scheme which weights timepoints depending upon their position in the window), such that our calculations are performed on a submatrix of the full data matrix.

There are three parameters to consider when computing DMD and/or ICA modes within a sliding window: *T*, the length of each window; *dT*, the number of timesteps by which to slide the window; and *r*, the number of modes to compute within each window. We observed that our results are relatively robust to these parameters. Within the results shown in the main manuscript, we have chosen *T* = 32, *dT* = 4, and *r* = 8. In seconds, this corresponds to roughly 22.4 s windows which are translated in increments of about 2.8 s. As discussed in section 4.3, oscillatory DMD modes appear in conjugate pairs, and thus ≤ *r* = 8 modes will be extracted within each window. For example, the gDMD set used in Figure 4 (the modes calculated from the REST1_LR scans of 120 subjects) consists of 160,756 total modes.

### 4.6. Mode Visualization

DMD modes are computed on the full 59,412-dimensional grayordinate space. However, this space is unnecessarily large for visualization purposes, for which we primarily want to visualize the macroscopic structure of individual modes. It is also disadvantageous for the clustering of modes; we want to cluster modes based upon their overall structural similarity, and applying some method of spatial smoothing is helpful in minimizing spurious, noise-driven correlations. For visualization and clustering purposes, we therefore bin the modes, computing the average magnitude of grayordinates within a particular patch of space.

Each grayordinate can be mapped onto a 3*D* coordinate in space. From this information, we first classify all grayordinates as on the left/right cortex and on the lateral/medial side. Each of these groups of grayordinates are then projected onto the sagittal plane and divided into 40 bins in each direction. This choice of bin granularity is arbitrary but was chosen heuristically to yield good visualization and clustering performance. Bins which contain no grayordinates are discarded, resulting in a total of 3,856 bins.

Given a 59,412-dimensional DMD mode **ϕ**_*j*_, we may average over the grayordinates within each bin to yield a 3,856-dimensional binned mode *m*_*j*_. All modes visualized within this paper have been binned in this fashion. Furthermore, modes have been binned before being clustered or correlated against each other, which was seen to substantially improve the performance of clustering.

### 4.7. Hierarchical Clustering

Having performed sliding window DMD mode extraction on the full set of 120 individuals, we wish to cluster modes based upon their spatial similarity on both the single-subject and whole-group level. In both cases we use the same hierarchical clustering pipeline, described as follows, applied to either the set of modes extracted from a single scan (the sDMD case) or from the full set of scans (the gDMD case).

A few pre-processing steps were found to be helpful in generating clean, robust clusters. First, modes are spatially binned as described in section 4.6. This reduces the dimensionality of modes for clustering purposes, averaging over noise while preserving larger-scale spatial structure. We then wish to filter out modes which lack large-scale continuous spatial structure. A quick heuristic for the spatial continuity of a mode is calculated and thresholded upon as follows: for each binned mode, we count the number of bins with a z-score ≥ 2 which also have a neighbor with z-score ≥ 2. Thresholding on *c* keeps only those modes which possess a certain level of spatial continuity (e.g., in Figure 4 we cluster only modes with *c* ≥ 25).

To group this filtered set of modes into a small set of interpretable clusters, we use hierarchical clustering as implemented in SciPy 1.0.0 (Jones et al., 2001). There are many metrics of spatial similarity which can be used in such a procedure. Qualitatively, we found the best performance (as indicated by the formation of tight, discrete clusters which group modes with similar large-scale spatial structure) by thresholding modes at a defined z-score, *z*_*t*_, to generate spatial masks [in Figure 4 we use *z*_*t*_ = 2.5; the clustering procedure is fairly robust to the choice of *z*_*t*_, as demonstrated in Appendix D (Supplementary Material)]. These masks were then clustered based upon the average spatial correlation within a group of modes.

We then form flat clusters that have a cophenetic distance of no greater than a defined threshold *t* (i.e., using the “distance” threshold option of the scipy.cluster.hierarchy function fclust). Figure 4 shows the clusters which are formed using *t* = 0.955, whereas Figure 5 shows finer-grained subclusters formed using *t* = 0.950. The choice of *t* will affect the size and granularity of the cluster assignment, and can be varied to obtain smaller subclusters of any particular cluster [the robustness of clustering to the choice of *t* is also explored in Appendix D (Supplementary Material)]. Note that in Figures 4, 5 we have only visualized clusters containing a minimum number of modes (≥ 400 in the gDMD case of Figure 4, and ≥ 5 for the sDMD case of Figure 5).

We compare these modes against a set of canonical RSNs, using the parcellation of Gordon et al. (2016). In Figure 5B we show the correlation of each mode against each of these canonical masks, where the ordering of the modes has been defined by the hierarchical clustering. The clustering succeeds at grouping modes which resemble similar RSNs, and many of the clusters appear to correspond to distinct networks: for instance, the bottom-rightmost cluster (cluster number 13) strongly corresponds to the “SMmouth” network. When the average of the modes within each cluster is plotted in Figure 4D, it indeed has the expected spatial structure.

### 4.8. Subject-Level DMNs

The subject-level Default Mode Networks (DMNs) shown in Figure 6 are found by calculating the sDMD clusters for modes from a particular subject, and taking the average of the cluster which most resembles the DMN. The optimal choice of *z*_*t*_ and *t* will vary from scan to scan; we therefore perform the clustering procedure several times for values of *z*_*t*_ ∈ (1.5, 3.0) and *t* ∈ (0.65, 0.98), saving the output cluster average which has the highest correlation with the gICA result. Though the choices of *z*_*t*_ and *t* afford relatively little control over the final spatial structure of the result, we must be mindful of the fact that we are selecting the best-performing result out of several different runs. For a fair comparison, we afford ICA the same opportunity: allowing the number of modes to vary in the range *r* ∈ (8, 10, 12, 16), calculating ICA multiple times, and saving only the result which correlates most highly with the gICA result. This was seen to be necessary, as the output of the ICA algorithm is not deterministic and will sometimes yield a low-quality result (often yielding correlations of ~0.1, as in the long tail of Figure 6B).

Each of these approaches were compared against the group-ICA and dual regression approach, the results of which are calculated and provided for download by the HCP (Filippini et al., 2009). This approach first calculates group-ICA, which is ICA performed on the temporally-concatenated full dataset of scans from 1,200 individuals. This calculates high-quality, population-level averaged gICA modes. The gICA mode corresponding to the DMN is then regressed against the scans for a single individual, to calculate an approximate timecourse of DMN activation within a particular individual's scans. There is then a second regression step of the timecourses of all spatial coordinates against the averaged-DMN timecourse. This yields a spatial map of coordinates which have similar time dynamics to the population-level DMN mode, which in effect yields a subject-level spatial map of the DMN. This results in clean, high-quality maps as seen in Figure 6, but has the drawback of requiring a reference mask calculated from the entire set of population data.

Figure 6B plots how strongly each subject-level ICA/sDMD DMN correlates with both (i) the full set of gICA modes, and (ii) the gICA mode calculated for the same subject. These are calculated by thresholding the modes at a z-value of *z* = 2 and then calculating the correlation coefficients between the thresholded masks.

### 4.9. Characterizing Cluster Dynamics

Assigning time dynamics to individual clusters (as in Figure 5B) follows trivially from the windowing process: each cluster consists of a collection of modes, each calculated in a particular window corresponding to a particular time. The time dynamics of a cluster are simply given by the times of the windows in which its constituent modes were found. Notably, this gives a binary measure of mode activation (a cluster either contains a mode within a particular time window or it doesn't). This is distinct from other methods of assessing the temporal dynamics of modes, such as the common technique of correlating a spatial mode with each frame of a scan; such techniques yield a continuous measure of mode activation, which does not fully disambiguate the activity of a mode from that of overlapping spatial patterns or random fluctuations. This disambiguation is accomplished in our approach through the assignment of modes into discrete clusters.

The gDMD pipeline was performed independently on the four sets of scans for all 120 individuals (the sets “REST1_LR,” “REST1_RL,” “REST2_LR,” and “REST2_RL”). This resulted in four sets of gDMD modes, which were correlated against each other to find the modes which were most similar across all sets. We chose the top six most consistent modes, as described in Appendix A (Supplementary Material), with the resulting modes visualized in Figure 7A. Each set of modes has associated time dynamics; as examples of how to characterize these dynamics we calculate both the occupancy matrix *O*_*ij*_ and transfer matrix *T*_*ij*_. The occupancy matrix *O*_*ij*_ indicates the number of windows in which both modes *i* and *j* are active. The diagonal elements *O*_*ii*_ count the total number of times mode *i* was active (with or without the presence of other modes). The transfer matrix *T*_*ij*_ shows the probability that if mode *i* is present, then mode *j* is present 30 s later.

We assess the reproducibility of our approach by comparing the occupancies of each cluster by each individual between different scans. This rests upon the assumption that if an individual shows increased activity in a particular cluster in one scan, they are then more likely to be more active in the same cluster within another scan. Note that we do not expect a perfect correlation near 1.0, but we do anticipate a significant one; for example, if an individual has a particularly active DMN in one scan, we may expect that same individual is more likely to have an active DMN in another scan, but not that the DMN should be active for the exact same amount of time. An example of such a comparison is plotted in Figure 8, where each point represents a different individual, and we do indeed observe a positive correlation. We correlated all combinations of the four scans against each other for all different modes, and report the median correlation and occupancy within the table of Figure 8. A significant correlation (in the sense that *p* < 0.05) is clear for all modes except for mode 4, indicating that our characterization of the dynamics indeed encodes underlying dynamic properties specific to different individuals.

## Data Availability Statement

Publicly available datasets were analyzed in this study. This data can be found here: https://db.humanconnectome.org.

## Author Contributions

All authors contributed to the conceptualization of the study. JK-G, DG, JK, and BB contributed to the mathematical conceptualization. KE and SR contributed to the domain-specific conceptualization. JK-G and BB developed the methodology, in consultation with JK and DG. KE and SR curated the data. JK-G wrote the software to perform the investigation and visualization. JK-G, SR, and BB supervised the project. All authors contributed to the writing of the manuscript.

### Conflict of Interest

The authors declare that the research was conducted in the absence of any commercial or financial relationships that could be construed as a potential conflict of interest.
