# Supplementary material for: Extracting Reproducible Time-Resolved Resting State Networks Using Dynamic Mode Decomposition
Source: Front Comput Neurosci. 2019 Oct 31;13:75. doi: 10.3389/fncom.2019.00075 (PMC6834549; doi:10.3389/fncom.2019.00075)
Supplement: Supplementary file 1 [file Data_Sheet_1.PDF]

Appendix A Comparing Clusters Across Scans

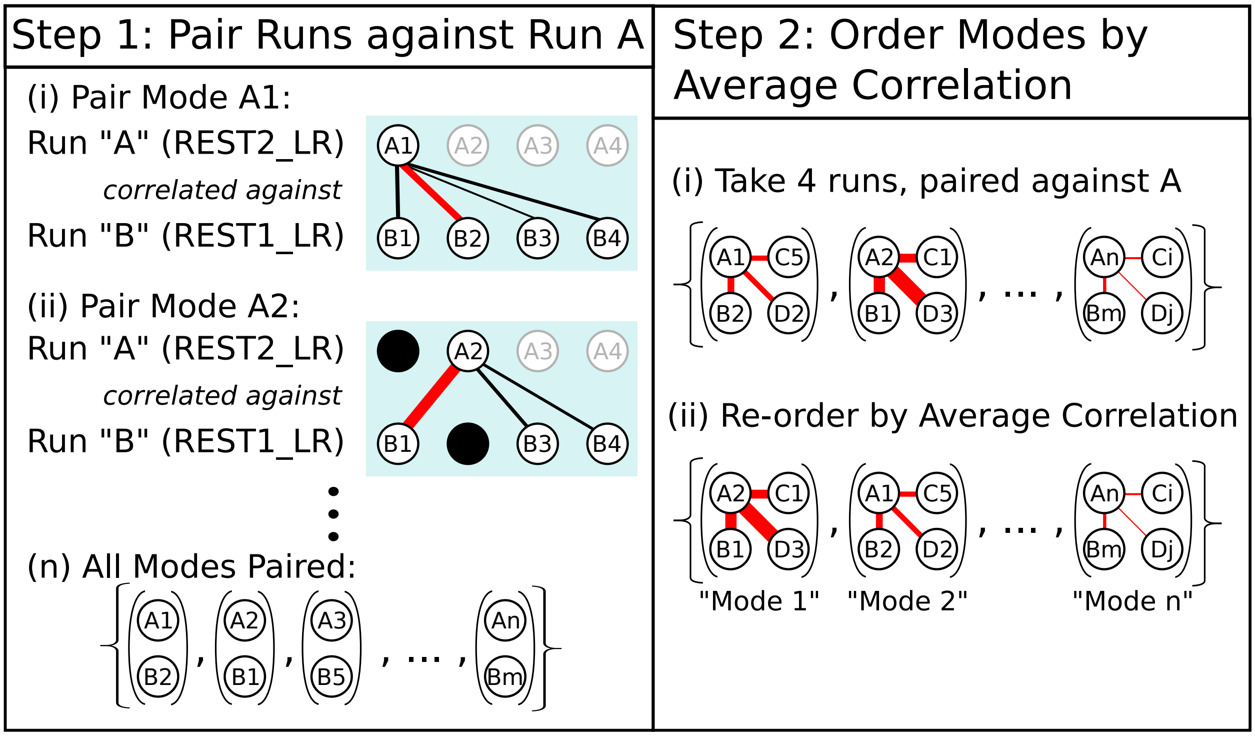

Figure 1: Procedure for pairing in ordering modes between different runs. When we process the four runs independently with identical parameters, they result in a different number of clusters, and thus a different number of corresponding averaged modes. Here we label the runs A, B, C, D; in the data, these refer to REST2_LR, REST1_RL, REST2_RL and REST1_LR, which yielded 36, 48, 49 and 42 modes respectively. We choose the run with the least number of modes (REST2_LR) as Run A, and match its modes to a unique mode from each of the other runs. **Step 1:** Iterate through the modes of Run A and match them (without replacement) to the most highly spatially correlated mode from Run B, until all modes are paired. Repeat this process for Runs C and D. **Step 2**: Calculate the average correlation between the A modes and the paired B/C/D modes, and re-order the modes by their average correlation. The modes which have the highest average correlation (i.e. which appear, on average, most similar between runs) we dub the "most consistent". Spatial correlations of the B/C/D modes against the paired A modes in our results are plotted in Figure 2.

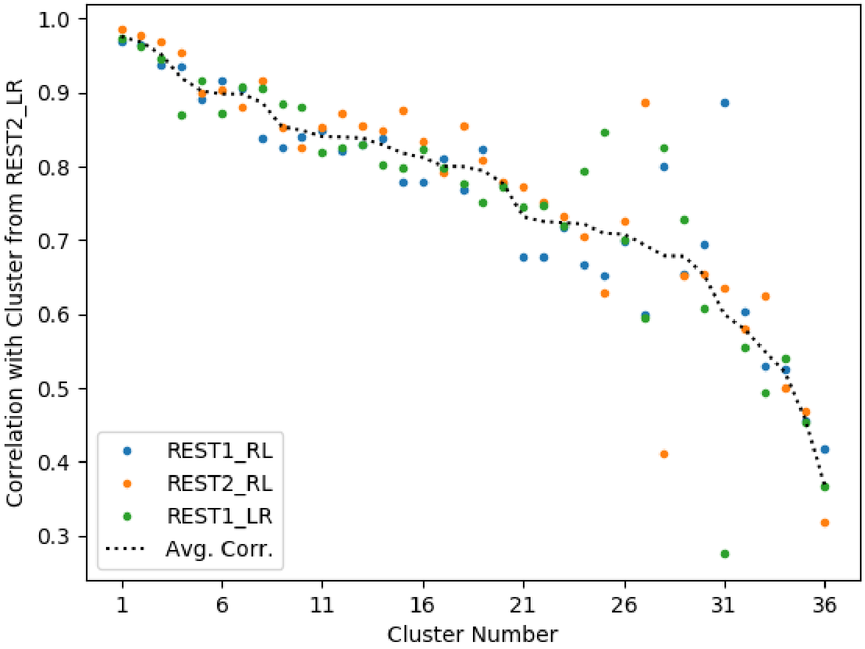

Figure 2: The averaged modes from each of the clusters from all four runs were paired together as described in Figure 1. As described there, these cluster-average modes are ordered by the average spatial correlation with those from the run REST2_LR. In the main manuscript, we visualize and analyze the dynamics of the top 6 modes, though this cut-off is arbitrary: similar spatial consistency appears in higher-numbered modes. For completeness, we include visualizations of the averaged modes of all clusters for all four runs on the following pages.

Appendix A.1 Averaged Modes from All Clusters

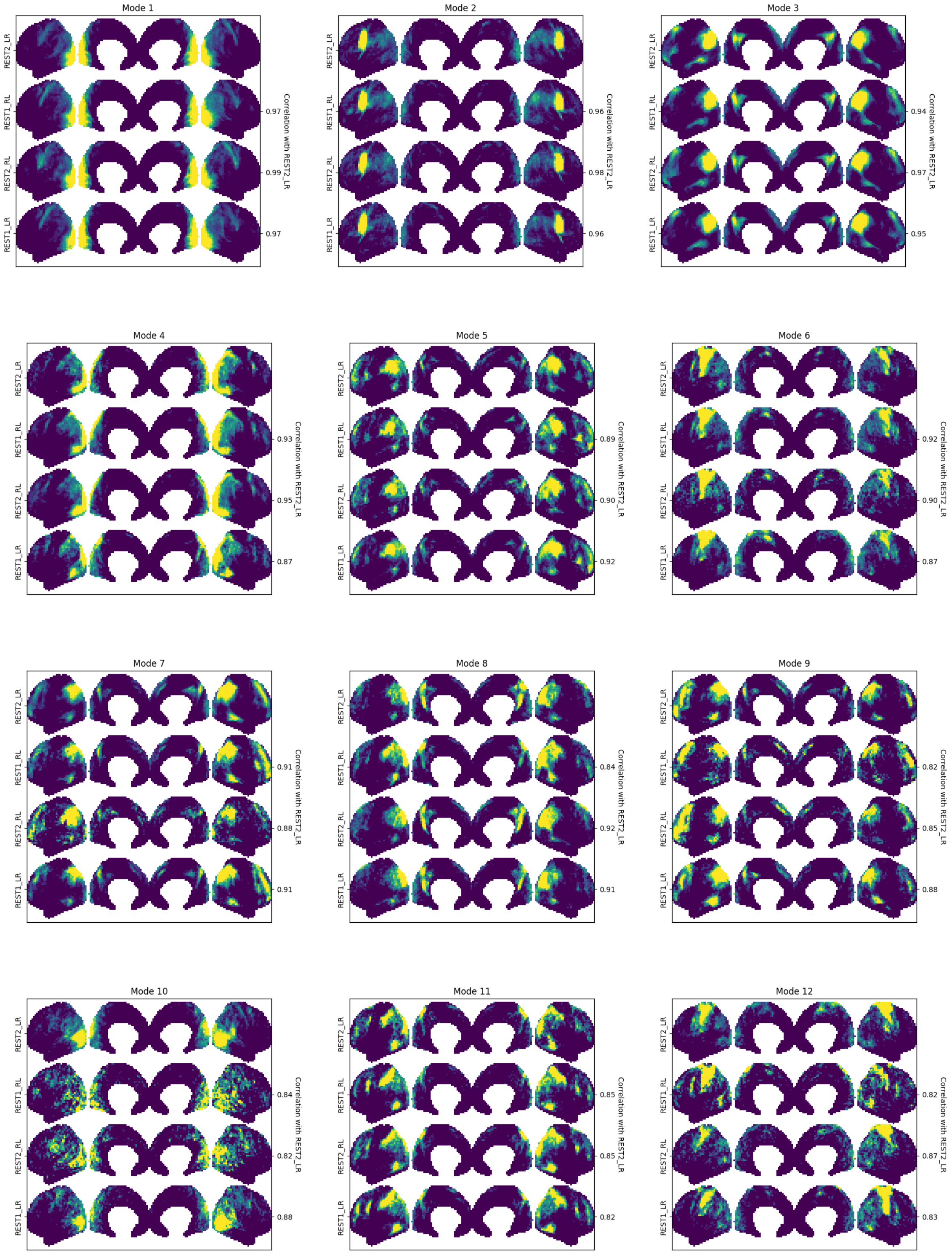

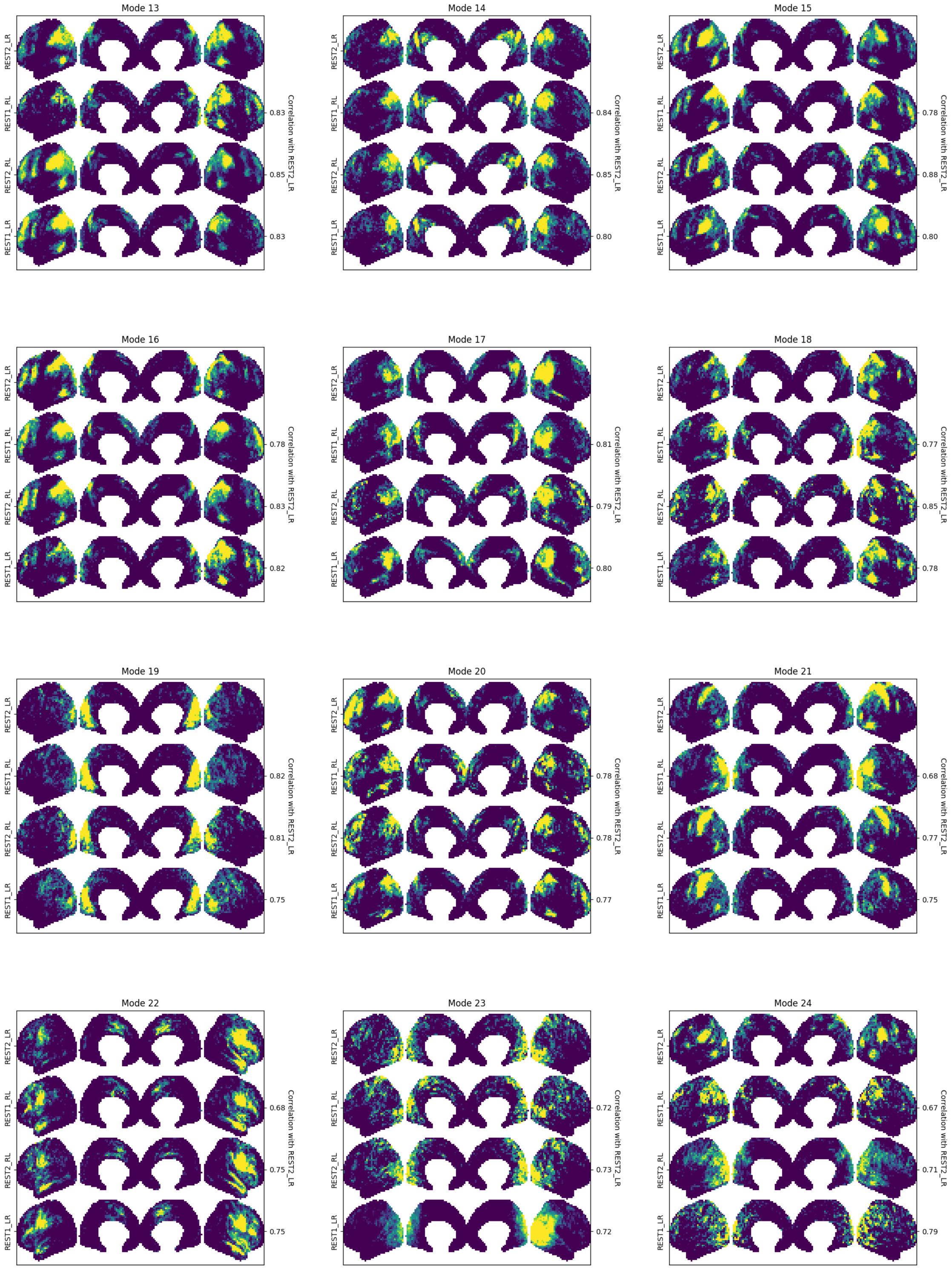
Mode 13
REST2_LR
REST1_RL
REST2_RL
REST1_LR
0.83
0.85
0.83
Correlation with REST2_LR
Mode 14
REST2_LR
REST1_RL
REST2_RL
REST1_LR
0.84
0.85
0.80
Correlation with REST2_LR
Mode 15
REST2_LR
REST1_RL
REST2_RL
REST1_LR
0.78
0.88
0.80
Correlation with REST2_LR
Mode 16
REST2_LR
REST1_RL
REST2_RL
REST1_LR
0.78
0.83
0.82
Correlation with REST2_LR
Mode 17
REST2_LR
REST1_RL
REST2_RL
REST1_LR
0.81
0.79
0.80
Correlation with REST2_LR
Mode 18
REST2_LR
REST1_RL
REST2_RL
REST1_LR
0.77
0.85
0.78
Correlation with REST2_LR
Mode 19
REST2_LR
REST1_RL
REST2_RL
REST1_LR
0.82
0.81
0.75
Correlation with REST2_LR
Mode 20
REST2_LR
REST1_RL
REST2_RL
REST1_LR
0.78
0.78
0.77
Correlation with REST2_LR
Mode 21
REST2_LR
REST1_RL
REST2_RL
REST1_LR
0.68
0.77
0.75
Correlation with REST2_LR
Mode 22
REST2_LR
REST1_RL
REST2_RL
REST1_LR
0.68
0.75
0.75
Correlation with REST2_LR
Mode 23
REST2_LR
REST1_RL
REST2_RL
REST1_LR
0.72
0.73
0.72
Correlation with REST2_LR
Mode 24
REST2_LR
REST1_RL
REST2_RL
REST1_LR
0.67
0.71
0.79
Correlation with REST2_LR

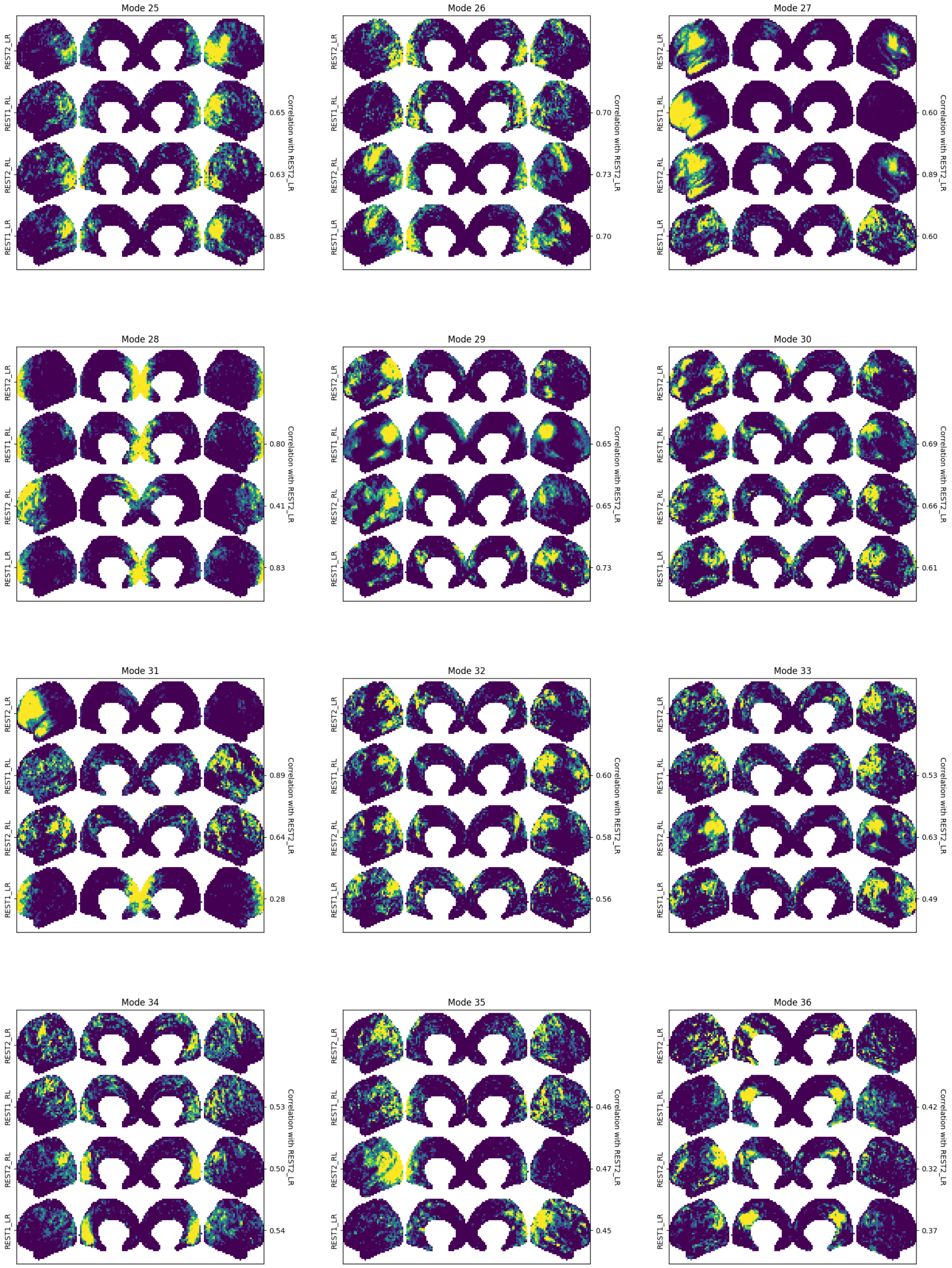
Mode 25
REST2_LR
REST1_RL
REST2_RL
REST1_LR
0.65
0.63
0.85
Correlation with REST2_LR
Mode 26
REST2_LR
REST1_RL
REST2_RL
REST1_LR
0.70
0.73
0.70
Correlation with REST2_LR
Mode 27
REST2_LR
REST1_RL
REST2_RL
REST1_LR
0.60
0.89
0.60
Correlation with REST2_LR
Mode 28
REST2_LR
REST1_RL
REST2_RL
REST1_LR
0.80
0.41
0.83
Correlation with REST2_LR
Mode 29
REST2_LR
REST1_RL
REST2_RL
REST1_LR
0.65
0.65
0.73
Correlation with REST2_LR
Mode 30
REST2_LR
REST1_RL
REST2_RL
REST1_LR
0.69
0.66
0.61
Correlation with REST2_LR
Mode 31
REST2_LR
REST1_RL
REST2_RL
REST1_LR
0.89
0.64
0.28
Correlation with REST2_LR
Mode 32
REST2_LR
REST1_RL
REST2_RL
REST1_LR
0.60
0.58
0.56
Correlation with REST2_LR
Mode 33
REST2_LR
REST1_RL
REST2_RL
REST1_LR
0.53
0.63
0.49
Correlation with REST2_LR
Mode 34
REST2_LR
REST1_RL
REST2_RL
REST1_LR
0.53
0.50
0.54
Correlation with REST2_LR
Mode 35
REST2_LR
REST1_RL
REST2_RL
REST1_LR
0.46
0.47
0.45
Correlation with REST2_LR
Mode 36
REST2_LR
REST1_RL
REST2_RL
REST1_LR
0.42
0.32
0.37
Correlation with REST2_LR

Appendix B Cluster Frequency Content

In the previous section, we paired clusters extracted from four different runs and then averaged the spatial modes in each of the clusters. Spatially, the average mode of each cluster appears quite similar across the four different runs. DMD extracts a temporal frequency to accompany each spatial mode, and thus each cluster also has a distribution of frequencies. The distribution of frequencies in each cluster in each of the four runs in shown in Figure 1.

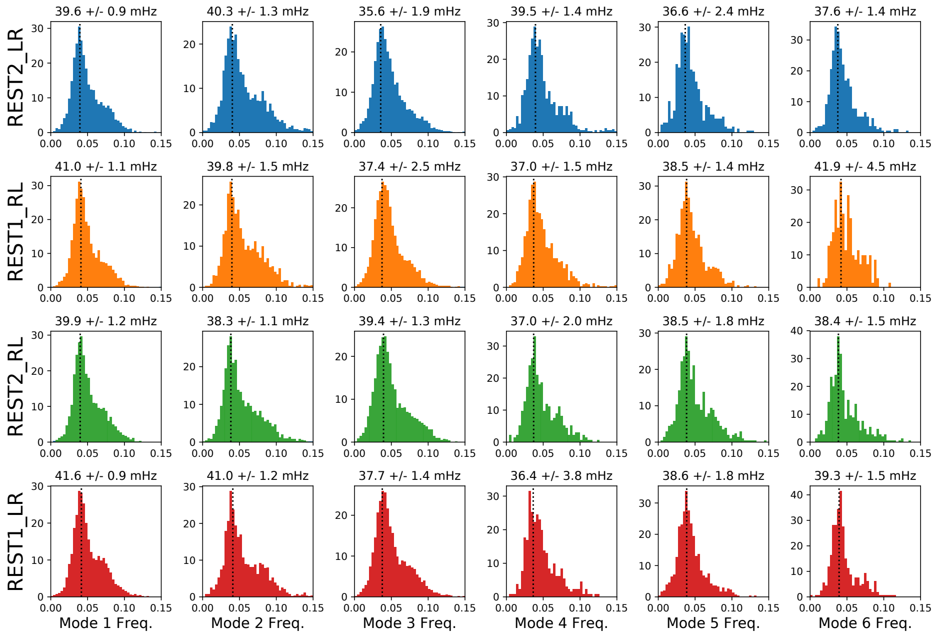

Figure 1: Frequency distributions for the modes constituting each of the six clusters in each of the four runs. Distributions appear approximately bimodal with a maximum-likelihood (peak) frequency at approximately 0.04Hz. The peak frequency (and its uncertainty) are approximated as described in this section, and are shown for each cluster.

The frequency distributions in Figure 1 are approximately bimodal; they are clearly not Gaussian, but appear to be well-approximated by the sum of two Gaussians, the first with a central frequency of ~ 0.04Hz and the second, shorter Gaussian with a central frequency of ~ 0.075Hz. It would be un-informative for these distributions to simply compute and compare the means. Instead, we fit a Gaussian Mixture Model to the frequency data using the `scikit-learn` module `mixture`. We select the optimal number of components using the Akaike information criterion, and take the maximum likelihood of the fit distribution. We perform this fitting procedure 100 times for each cluster on randomly-selected subsets of 75% of the data, and take the mean and standard deviation of these 100 trials as our estimate of the peak frequency and its uncertainty. This estimate of the peak frequency and its uncertainty are plotted for each mode in Figure 2

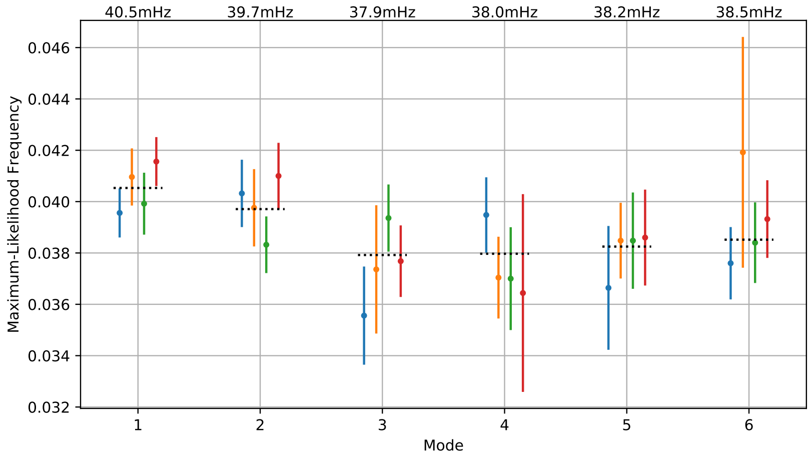

Figure 2: Peak frequencies inferred from the frequency distributions in Figure 1. For each mode, the peak was estimated for 100 random subsets of 75% of the data, and the resulting mean and standard deviation are plotted here. The dashed black lines indicate the mean frequencies of each cluster across the four runs. In short, the estimated peak frequencies are too imprecise to yield definitive conclusions, but these preliminary results suggest that the frequency content of different modes may vary reproducibly.

The distributions in Figure 1 are broad, and the estimates of peak frequency in Figure 2 are subsequently imprecise. This makes it difficult to make definitive statements about how the frequency content of the clusters are reproducibly varying between different scans. However, this preliminary analysis suggests possible reproducible variation: for example, Modes 1 and 2 appear to reliably have a peak frequency of close to 0.040Hz, whereas Modes 3-5 have a slightly lower peak frequency of 0.038Hz.

These preliminary analyses leave several open questions: does each network (corresponding to each cluster) have a distinct and reproducible frequency distribution? Does the peak frequency of each network vary from individual to individual, and could this individual variation in RSN frequency serve as a useful biomarker? In this paper we use the "exact DMD" algorithm for computing DMD, which is computationally efficient but yields a biased and imprecise estimate of the frequency. This has led to methods such as Optimized DMD [1], which considerably improves the precision of the frequency estimate but is much more computationally expensive. Future work should consider the use of these more expensive methods, as they may be able to characterize RSN frequency to a level of precision such that RSN frequency variations could be characterized down to the level of single subjects.

[1] T. Askham and J.N. Kutz. "Variable projection methods for an optimized dynamic mode decomposition." arXiv preprint arXiv:1704.02343, 2017.

Appendix C Singular Value Spectrum

In calculating windowed DMD, we need to select the number of modes to extract from each window. Throughout the paper we have chosen $n = 8$ modes to be extracted from each of our 32-frame windows. The appropriateness of this choice can be investigated by looking at the distribution of singular values. In this section, we look at the singular values from all the windows from a single scan (Subject 102513, run REST1_LR). DMD is performed over 32-frame windows, slid over a 1200-timestep scan in increments of 4-frames, leading to 293 windows. DMD is thus performed 293 times, including 293 SVD computations. The distributions of singular values from each of these computations is shown in Figure C1.

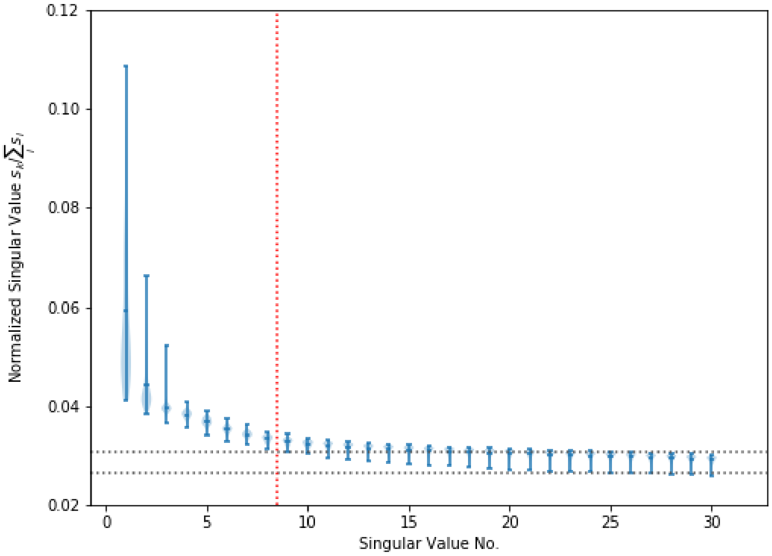

Figure C1: Distributions of singular values over the 293 windows of a single scan (Subject 102513, run REST1_LR). Our cutoff includes the first 8 modes (indicated by the dotted red line), which sums to only $\sim$ 40% of the energy as there is a large amount of noise. We roughly approximate a "noise floor" for singular values by taking the average minimum/maximum singular values of Modes 20-30, yielding the range indicated by the black dotted lines in the figure. Figure C2 shows a zoomed version of this figure, to better display the distributions close to our $n = 8$ cutoff.

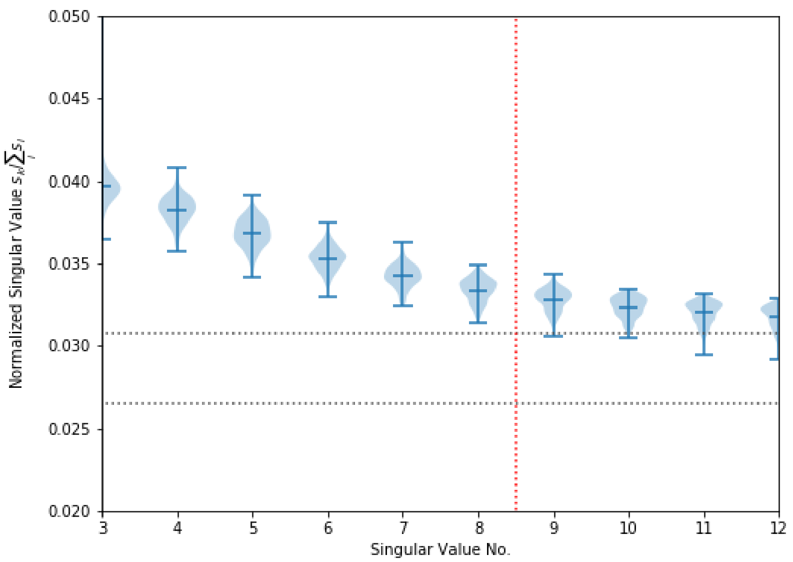

Figure C2: The same as Figure C1, zoomed in around the $n = 8$ cutoff. After this cutoff, singular values begin to dip into the "noise floor" range. This suggests that $n = 8$ is a reasonable choice, but also suggests that similar nearby choices of n should yield similar results, as modes in this range carry only a small (though potentially non-negligible) amount of energy. Indeed, we find that our results are not particularly sensitive to the precise choice of n.

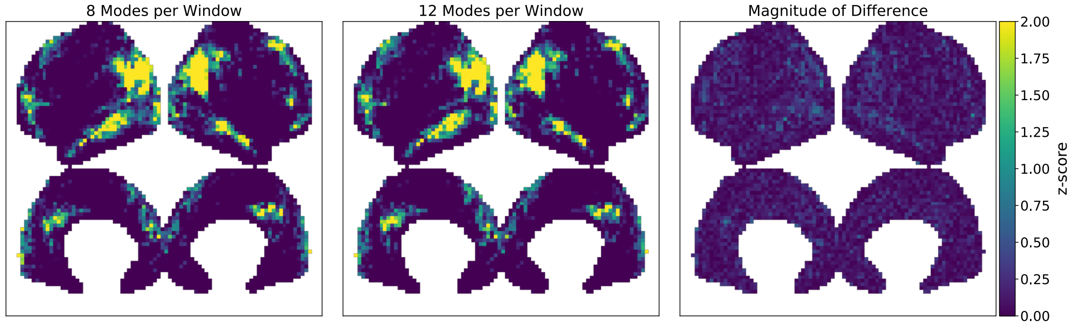

Figure C3: The Default Mode Network computed for Subject 102513, using the method of Section 2.4 in the main text. The computation was identical except that a different cutoff was used: $n = 8$ modes were extracted from each window for the first panel, and $n = 12$ were extracted for the second panel. The third panel shows the magnitude of the difference between these results (plotted on the same colorscale), which is barely visible. This corroborates the claim that the truncation drops modes which are mostly noise and do not carry an appreciable amount of a signal, and that this truncation therefore does little to affect the final result.

Appendix D Robustness to Clustering Parameters

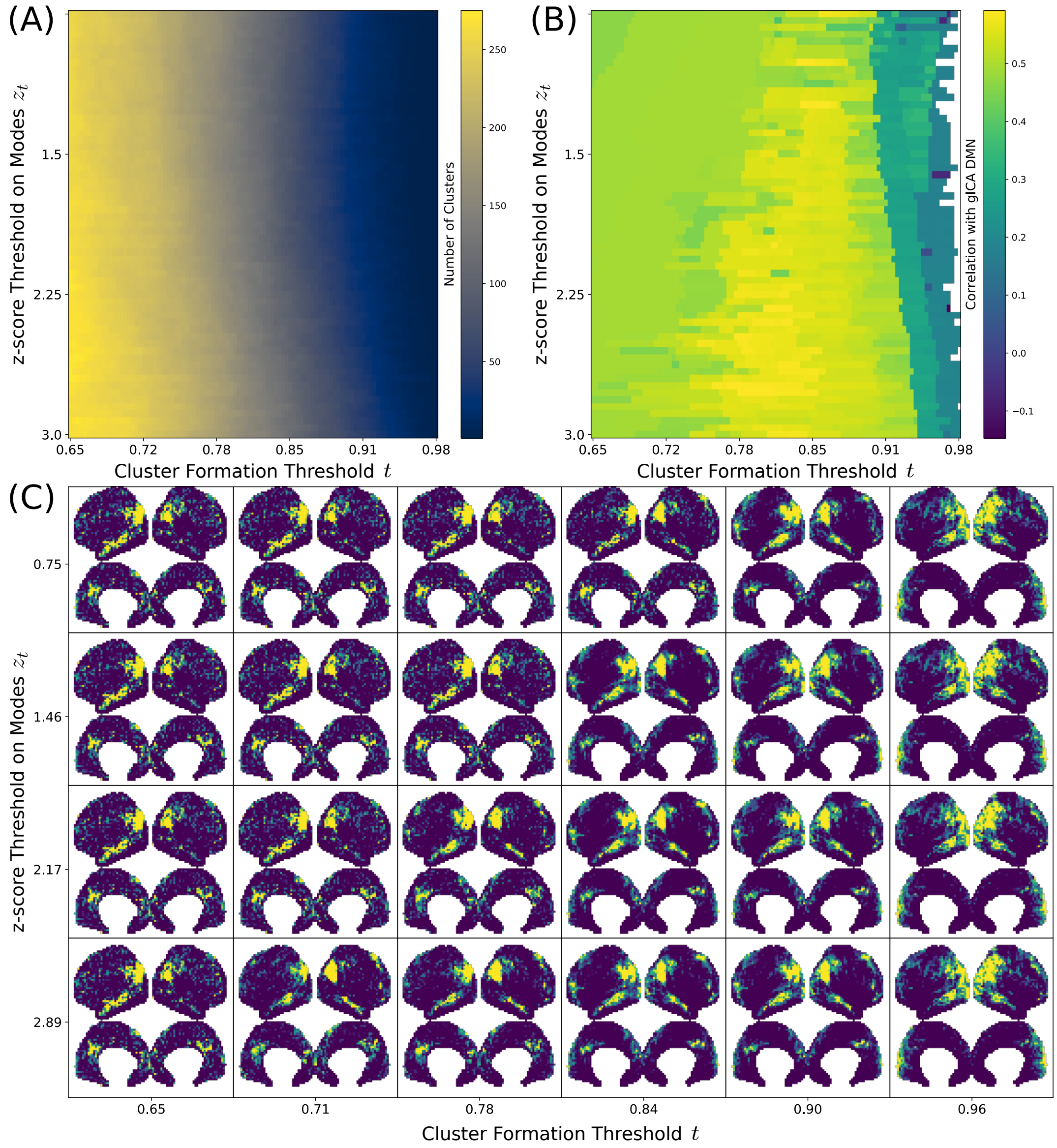

Figure D1: The sDMD analysis of Figure 6 (Subject 102513) was repeated on a grid of clustering parameters: the pre-clustering mode threshold z_t, and the cluster formation threshold t (see Section 4.7). **(A)** The total number of clusters. **(B)** Correlation between the gICA DMN and our result. This is optimal when z_t is chosen to form a moderate number of clusters. **(C)** The DMN at various parameter values. The overall structure remains similar (except for the limit of high t, in which other RSN clusters are merged together).

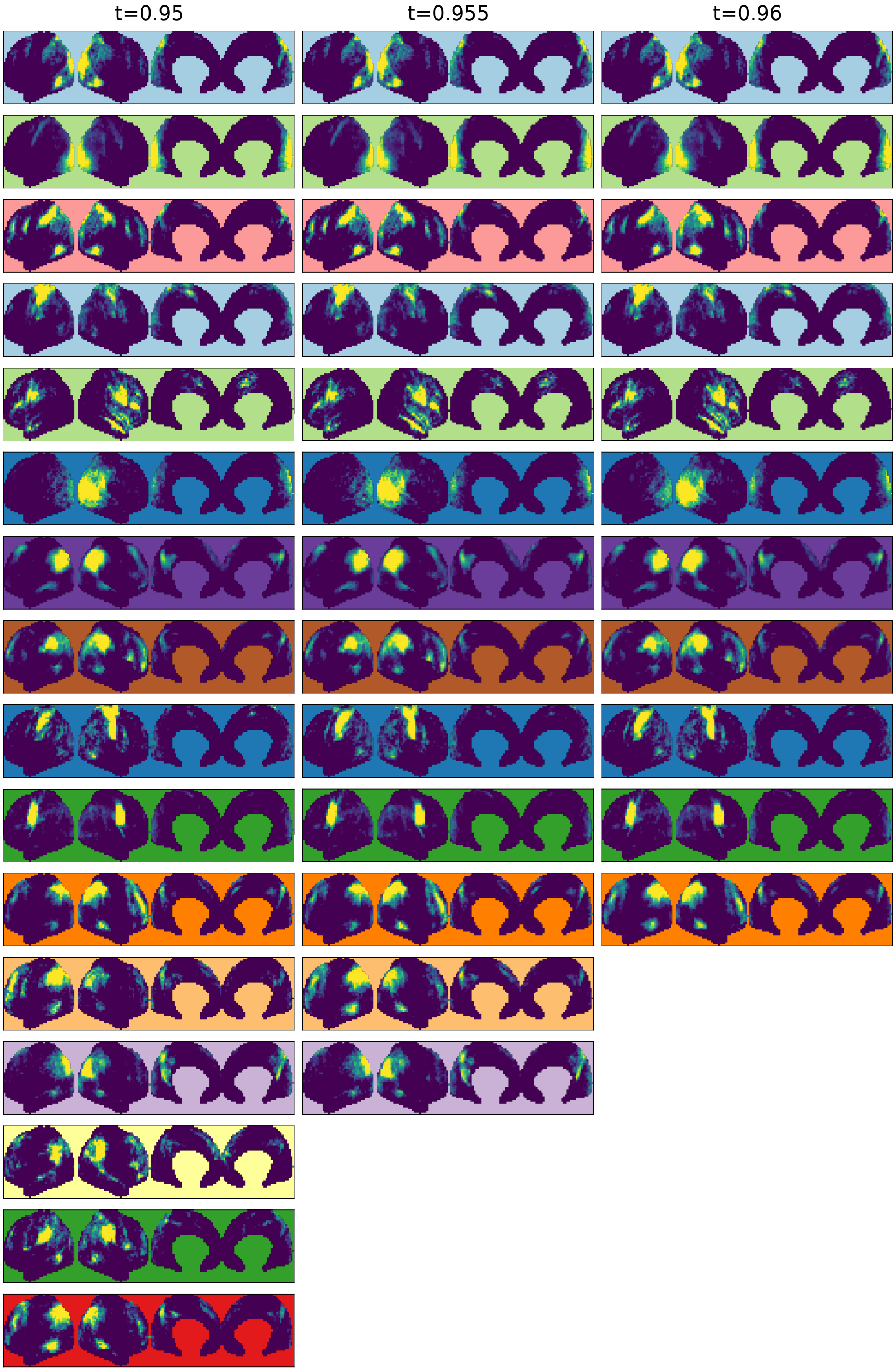

Figure D2: The gDMD pipeline of Figure 4 was repeated for varying choices of the cluster formation threshold t. The main text uses a value of $t = 0.955$, and changing this by ± 0.005 has the expected result: decreasing/increasing the threshold breaks the modes into more/fewer clusters. Most of the clusters are nearly identical, but the effect is most clear in the DMN-like clusters near the bottom. As the threshold is increased, these modes are consolidated into fewer, larger clusters.
